# Comparative Analysis of Salivary Gland Proteomes of Two *Glossina* Species that Exhibit Differential Hytrosavirus Pathologies

**DOI:** 10.3389/fmicb.2016.00089

**Published:** 2016-02-09

**Authors:** Henry M. Kariithi, İkbal Agah İnce, Sjef Boeren, Edwin K. Murungi, Irene K. Meki, Everlyne A. Otieno, Steven R. G. Nyanjom, Monique M. van Oers, Just M. Vlak, Adly M. M. Abd-Alla

**Affiliations:** ^1^Biotechnology Research Institute, Kenya Agricultural and Livestock Research OrganizationNairobi, Kenya; ^2^Insect Pest Control Laboratory, Joint FAO/IAEA Programme of Nuclear Techniques in Food and Agriculture, International Atomic Energy AgencyVienna, Austria; ^3^Laboratory of Virology, Wageningen UniversityWageningen, Netherlands; ^4^Department of Medical Microbiology, Acıbadem Universityİstanbul, Turkey; ^5^Laboratory of Biochemistry, Wageningen UniversityWageningen, Netherlands; ^6^South African National Bioinformatics Institute, University of the Western CapeCape Town, South Africa; ^7^Department of Biochemistry, Jomo Kenyatta University of Agriculture and TechnologyNairobi, Kenya

**Keywords:** SGH syndrome, asymptomatic infection, LC-MS/MS, pathogenesis, *Hytrosaviridae*, hypertrophy, unfolded protein response

## Abstract

*Glossina pallidipes* salivary gland hypertrophy virus (GpSGHV; family *Hytrosaviridae*) is a dsDNA virus exclusively pathogenic to tsetse flies (Diptera; Glossinidae). The 190 kb GpSGHV genome contains 160 open reading frames and encodes more than 60 confirmed proteins. The asymptomatic GpSGHV infection in flies can convert to symptomatic infection that is characterized by overt salivary gland hypertrophy (SGH). Flies with SGH show reduced general fitness and reproductive dysfunction. Although the occurrence of SGH is an exception rather than the rule, *G. pallidipes* is thought to be the most susceptible to expression of overt SGH symptoms compared to other *Glossina* species that are largely asymptomatic. Although *Glossina* salivary glands (SGs) play an essential role in GpSGHV transmission, the functions of the salivary components during the virus infection are poorly understood. In this study, we used mass spectrometry to study SG proteomes of *G. pallidipes* and *G. m. morsitans*, two *Glossina* model species that exhibit differential GpSGHV pathologies (high and low incidence of SGH, respectively). A total of 540 host proteins were identified, of which 23 and 9 proteins were significantly up- and down-regulated, respectively, in *G. pallidipes* compared to *G. m. morsitans*. Whereas 58 GpSGHV proteins were detected in *G. pallidipes* F_1_ progenies, only 5 viral proteins were detected in *G. m. morsitans*. Unlike in *G. pallidipes*, qPCR assay did not show any significant increase in virus titers in *G. m. morsitans* F_1_ progenies, confirming that *G. m. morsitans* is less susceptible to GpSGHV infection and replication compared to *G. pallidipes*. Based on our results, we speculate that in the case of *G. pallidipes*, GpSGHV employs a repertoire of host intracellular signaling pathways for successful infection. In the case of *G. m. morsitans*, antiviral responses appeared to be dominant. These results are useful for designing additional tools to investigate the *Glossina*-GpSGHV interactions.

## Introduction

*Glossina pallidipes* salivary gland hypertrophy virus (GpSGHV; family *Hytrosaviridae*) is a dsDNA virus whose 190 kb genome encodes more than 60 confirmed proteins (Abd-Alla et al., 2008, 2009b; Kariithi et al., 2013a). The *Hytrosaviridae* family consists of only one other member, the housefly *Musca domestica* (Diptera; Muscidae) hytrosavirus (MdSGHV; Coler et al., 1993). However, detection of hytrosavirus-like infection symptoms, i.e., the salivary gland hypertrophy syndrome (SGH) in the Narcissus bulb fly *Merodon equestris* (Diptera; Syrphidae; Amargier et al., 1979) and in male accessory gland filaments of the parasitic wasp *Diachasmimorpha longicuadata* (Hymenoptera; Braconidae; Luo and Zeng, 2010) implies that the *Hytrosaviridae* potentially contains other members. The intrinsic properties of hytrosaviruses, i.e., covert chronic infection of adult stages without expression of detectable SGH symptoms, have probably hindered the discovery of other *Hytrosaviridae* family members up until now. GpSGHV is exclusively pathogenic to the tsetse fly (Diptera; Glossinidae), the vector of a group of neglected tropical diseases called the African trypanosomiases (Mattioli et al., 2004). Research on GpSGHV pathobiology has been hindered by a lack of an *in vitro* cell culture system to support the virus replication (Abd-Alla et al., 2011a). Attempts to multiply GpSGHV in alternative insect hosts such as *M. domestica* have so far been unsuccessful. The only available method to multiply GpSGHV is via intra-hemocoelic injections of virus suspension in *G. pallidipes* (Kariithi et al., 2013b).

A mature GpSGHV virion contains four distinct structural components (nucleocapsid core, tegument, envelope, and helical surface projections) composed of 61 virally-encoded proteins (Kariithi et al., 2010). The GpSGHV virion also contains 51 host-derived cellular proteins: some are incorporated into the virus particles and may play roles in virus replication and transmission (Kariithi et al., 2013a,b). In *G. pallidipes*, GpSGHV is transmitted horizontally via saliva during feeding (Abd-Alla et al., 2010) and vertically (transovarial) via the fat body tracheal system and milk gland secretions (Boucias et al., 2013). GpSGHV infection in laboratory colonies of *G. pallidipes* can either be asymptomatic or symptomatic with the former being the most rampant in laboratory colonies of this tsetse species (Abd-Alla et al., 2010). However, the asymptomatic infection state can convert to a symptomatic state, leading to reproductive dysfunction and reduced fecundity in addition to SGH symptoms (Abd-Alla et al., 2007; Lietze et al., 2011; Boucias et al., 2013). More than 40% of salivary gland (SG) proteins appear to be specifically expressed in *G. pallidipes* flies with overt SGH symptoms but not in asymptomatic flies (Kariithi et al., 2011). Unlike in the laboratory tsetse fly colonies, GpSGHV infection is mainly covert (latent) in wild *G. pallidipes* populations. Occurrence of SGH symptoms have been reported in other *Glossina* species such as *G. m. morsitans* (Jura et al., 1993) and *G. m. centralis* (Sang et al., 1997). However, SGH symptoms are rare especially in species other than *G. pallidipes*. Notably, even in *G. pallidipes* the occurrence of SGH symptoms is an exception rather than the rule (Boucias et al., 2013). The pathobiology of GpSGHV in species other than *G. pallidipes* has not been so far investigated.

Whether naturally or artificially infected, the GpSGHV infection rate is low, but males are more susceptible to infections compared to females (Abd-Alla et al., 2007; Boucias et al., 2013). After acquisition through a blood meal, GpSGHV translocates to the SGs where it primarily replicates (Garcia-Maruniak et al., 2009). In *G. pallidipes*, intra-hemocoelic GpSGHV injection leads to significant increase in the viral titters in the whole fly, but the injected virus is not released via saliva during feeding and there is no development of overt SGH symptoms (Boucias et al., 2013). Rather, SGH symptoms are overt in the F_1_ progenies of the infected mothers. It is yet to be confirmed in which host tissues GpSGHV replicates after artificial injection. However, the current school of thought is that in naturally-infected *G. pallidipes*, the virus replicates in the male reproductive accessory glands (Sang et al., 1999) and the gut (Sang et al., 1997) but without any teratogenic effects.

The pathological, morphological and ultrastructural effects of GpSGHV infection in *G. pallidipes* SGs have been studied to considerable length (Kariithi et al., 2011, 2013a; Guerra et al., 2013). However, no such studies have been performed in other *Glossina* species. Further, the molecular basis for the differential GpSGHV pathology in different *Glossina* species is still unclear. Here, we investigated GpSGHV-induced modulation of total protein expression in the SGs of *G. pallidipes* and *G. m. morsitans*, with special emphasis on the host pathways that are potentially employed by the virus during infection. We hypothesized that GpSGHV infection in *Glossina* is under the control of host-and/or virus-encoded factors (proteins/peptides) whose interactions influence the expression or lack of overt SGH symptoms. We tested the hypothesis by comparing the SG proteomes of GpSGHV-infected vs. mock-infected *G. pallidipes* and *G. m. morsitans* flies. The host (and viral) proteins identified in this study are potential targets for control of GpSGHV infections in tsetse fly mass production facilities. For instance, antiviral strategies could be developed to block virus replication and egress (Esfandiarei et al., 2006; Cheshenko et al., 2010; Chen et al., 2011), prevent the establishment of virus replication complexes (Saxena et al., 2012) and prevent development of cellular proliferation (Guergnon et al., 2011). Such antiviral approaches are applicable in the control of virus infections in mass production of other insects.

## Materials and methods

### Tsetse flies

The *G. m. morsitans* and *G. pallidipes* flies used in this study were obtained from a colony maintained at the Joint FAO/IAEA Insect Pest Control Laboratories (IPCL), Seibersdorf, Austria. For each treatment described below, groups of experimental flies were kept in holding cages (diameter of 20 cm and height of 5 cm) at a density of 75 flies per cage and a mating ratio of 1:4 (male: female). The holding cages had netting on top and bottom for fly feeding and pupae collection, respectively. The experimental flies were reared at 23 ± 1°C, 75–80% relative humidity, 12 h scotophase and fed on defibrinated bovine blood meals (15–20 min; 3 times per week; Feldmann, 1994). The pupae from the sequential larviposition cycles were collected and incubated at 24°C until eclosion of the adult F_1_ progenies. For further analyses, male F_1_ progenies were selected from the fourth larviposition cycle (G_4_) based on available data that the incidence of SGH symptoms reaches 100% at the G_4_ (Boucias et al., 2013). It should be noted that males were used because they are significantly more susceptible to expression of SGH symptoms than the females (Abd-Alla et al., 2007). To allow for development of SGH symptoms, the selected F_1_ male progenies were reared for 4 weeks (equivalent to 12 blood meals) under the same insectaria conditions and handled as the parents. All the treatments described here were replicated at least three times.

### Preparation of GpSGHV inoculum and injections of tsetse flies

To prepare the GpSGHV inoculum, one intact pair of SGs displaying overt SGH symptoms were dissected from an adult (10-day old) male *G. pallidipes* fly and stored in 1 ml of ice-cold sterile saline (pH 7.4). The SGs were then homogenized and clarified by brief centrifugation (500 × g; 3 min; 4°) to remove tissue debris. The supernatants were sterilized by passing through a 0.45-μm filter unit and the virus titters present in the filtrate were estimated by a quantitative polymerase chain reaction (qPCR) as described by Abd-Alla et al. (2009a). By this qPCR method, an average of 1 × 10^6^ virus copies were estimated to be present in a 2 μl aliquot of the virus preparation and was used for tsetse fly injections. For infections, teneral (newly eclosed; non-fed) female *G. m. morsitans* and *G. pallidipes* flies were artificially (intra-hemocoelic) injected with the virus preparations using a protocol described by Boucias et al. (2013). Briefly, the female flies selected from the colony as described above were inoculated with either 2 μl of the virus inoculum or 2 μl of filter-sterilized PBS (mock infections). Following the injections, the females were mated with asymptomatic males; these females were then separated from the males and subsequently maintained in the insectary until they produced the F_1_ progenies as described above.

### Detection of viral DNA in infected *G. pallidipes* and *G. m. morsitans* flies

To confirm GpSGHV infections, the 4 week-old F_1_ male progenies produced by the mock- and virus-infected mothers were screened using a diagnostic PCR protocol described by Abd-Alla et al. (2007). For this, genomic DNA was extracted from one intermediate excised leg of individual flies using DNeasy Tissue Kit (QIAGEN Inc., Valencia, CA). PCR amplifications were performed using primers and conditions previously described (Abd-Alla et al., 2007, 2011b), and the PCR products analyzed on 1% agarose gels. Flies were considered to be non-infected, moderately-infected or highly infected if there were no visible bands or showed faint bands or thick bands, respectively, on agarose gels as previously described (Abd-Alla et al., 2010). The actual virus copy numbers and the virus density levels were determined using qPCR essentially as described by Abd-Alla et al. (2009b). For virus density levels, the qPCR data were normalized against the tsetse β-*tubulin* gene (Wang et al., 2013). The samples with high virus infections (based on the agarose gels) were subsequently used for mass spectrometry measurements as virus-infected samples. This cut off was especially used in the case of *G. m. morsitans* flies, which do not usually show overt SGH symptoms. For *G. pallidipes*, samples with overt SGH symptoms (corresponding to samples with high infections from the agarose gels) were selected for mass spectrometry measurements. For negative controls, samples from 4-week-old male flies were selected from the mock-infected fly groups (confirmed to be PCR-negative). Ten flies from each of the replicated treatments described above were selected for subsequent SG dissections.

### Preparation of SG protein extracts

To prepare protein extracts, SGs from the F_1_ progenies described above were dissected 2 days after the flies had their last blood meals to allow for full digestions (Abd-Alla et al., 2009a). From each of the selected flies, intact pairs of SGs were individually dissected, during which the occurrence of SGH symptoms was assessed. The dissected SGs were preserved (at 4°C) in 150 μl sterile saline complemented with protease inhibitors (Roche Diagnostics, Germany). Then, each of the pool of 10 pairs of SGs were homogenized using a glass/Teflon homogenizer and ultra-sonicated (Sonifier cell disruptor, Branson, CT, USA) as described by Kariithi et al. (2013a). The homogenates were freeze-thawed and clarified three times by centrifugation (7500 × g; 10 min; 4°C). The supernatants were pooled and the proteins quantified using BCA Protein Assay (Bio-Rad) according to manufacturer's instructions. Then, equal quantities (600 ng) of the proteins (from each of the pooled 10 SGs per each of the three replicates) were electrophoresed using 12% SDS-PAGE gels (Invitrogen) as described by Green and Sambrook (2012). The gels were stained with colloidal CBB stain (NuPAGE Novex; Invitrogen). The middle sections of entire gel lanes were excised, and each of the gel lanes was divided into eight slices (equal portions) each of which was cut into approximately 1 mm^3^ pieces.

### Mass spectrometry and identification of SG proteins

To prepare peptides for mass spectrometry measurements, the gel slices containing the SG proteins were subjected to in-gel trypsin digestions as previously described (Kariithi et al., 2013a). Briefly, after washing the gel pieces with 50 mM ammonium bicarbonate (ABC) buffer and ABC buffer/50% (vol/vol) acetonitrile (ACN), proteins were reduced and alkylated using dithiothreitol and iodoacetamide, followed by washing with ABC/ABC-ACN buffers and trypsin digestions. Tryptic peptides were then analyzed by liquid chromatography-tandem mass spectrometry (LC-MS/MS; Lu et al., 2011). To identify the SG proteins, the MS/MS spectra obtained from the LC-MS/MS measurements were searched against a tsetse fly database, a GpSGHV database, a contaminant database containing sequences of common contaminants, and a decoy database constructed by reversing all protein sequences downloaded from UniProt. The MS searches were performed using MaxQuant v 1.3.0.5 (Cox and Mann, 2008) and Andromeda as the database search engine (Cox et al., 2011). A maximum false discovery rate (FDR) of less than 0.01 was set at the peptide and protein levels. MaxQuant search parameters included variable oxidation of M, fixed carboxamidomethylation of C, and extra variable modifications for de-amidation of N and Q. “Label-free quantification” (LFQ) and “match between runs” (set to 2 min) options were enabled. De-amidated peptides were allowed to be used for protein quantification.

### Quantification and characterization of SG proteins

To quantify the SG proteins, the resulting MaxQuant protein list was filtered to show only those proteins with a minimum of two peptides matching the same protein, of which at least one peptide was unique and unmodified. All other quantification settings were set at default. Any hits to the decoy sequences and hits with modified peptides only were deleted from the list of protein/peptides groups. To easily compare abundances of the same proteins between the controls and virus-infected samples, logarithms (Log_10_) of normalized LFQs were used. Logarithms of the total intensity corrected for the number of measurable peptides (i.e., intensity based absolute quantization; iBAQ) were used to compare the levels of different proteins from the same sample (mocks vs. GpSGHV-infected; Schwanhausser et al., 2011). Proteins were considered to be up- or down-regulated when their Log_10_ protein abundance ratios were larger or smaller than zero, respectively. Proteins were considered significantly up-regulated when their Log_10_ protein abundance ratios were larger than six. Gene Ontology annotation of the identified proteins were created using Blast2GO v 3.0.4 (Conesa et al., 2005). Analyses of the protein motif/domain were performed using various bioinformatics softwares, including SMART (Schultz et al., 1998) and InterProScan (Zdobnov and Apweiler, 2001).

## Results

### Detection of viral DNA in infected *G. pallidipes* and *G. m. morsitans* flies

**Figure 1:**
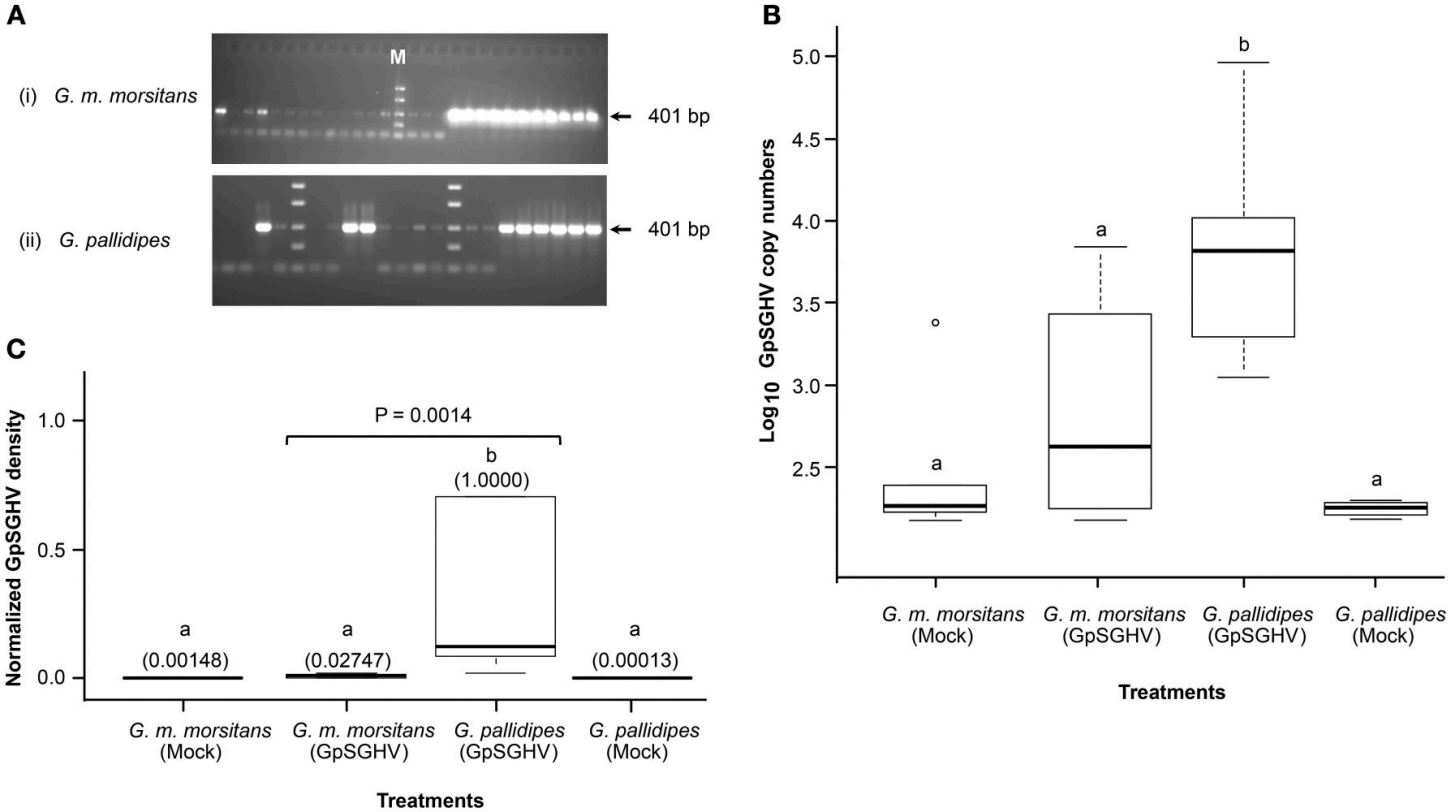
**Detection of GpSGHV infections in experimental flies**. **(A)** Sections of agarose gels used to analyze GpSGHV infections in virus-infected *G. m. morsitans* (i) and *G. pallidipes* flies (ii) using a diagnostic PCR protocol (Abd-Alla et al., 2007). Shown are non-infected samples (no bands), moderately-infected samples (faint bands) and highly infected samples (thick bands). M is molecular marker. The PCR amplifications were performed using genomic DNA extracted from single intermediate legs excised from 4-week old male F_1_ progenies produced by the control (Mock), or virus-infected (GpSGHV) flies. Determination of the GpSGHV copy numbers and the virus density levels by qPCR are shown in **(B,C)**, respectively. For determination of virus copy numbers **(B)**, 10-fold serially diluted viral DNA (targeting *odv-e66* gene) were used as internal standards as described by Abd-Alla et al., 2009a. For determination of the virus expression levels, qPCR data were normalized using a tsetse fly housekeeping gene (β-*tubulin*). Viral density levels in the virus-infected *G. pallidipes* progenies were significantly higher (*P* = 0.0014) than the levels in the virus-infected *G. m. morsitans* flies. The values in the parentheses **(C)** indicate the virus density levels. Letters *a* and *b* represent significant differences between the samples (i.e., there was no significant difference between samples labeled *a*, while *a* and *b* were significantly different).

We first analyzed GpSGHV replication in the F_1_ progenies from virus-infected mothers. We detected varying levels of GpSGHV infections in *G. m. morsitans* flies: non-infected, moderately infected and highly infected as evidenced by the absence of bands, faint bands and thick bands, respectively, in the agarose gels presented in the Figure 1A. The different GpSGHV infection levels we obtained for *G. m. morsitans* in Figure 1 were comparable to the results obtained for *G. pallidipes*, as well as results from our previous studies in *G. pallidipes* (compare with Figure 1 in Abd-Alla et al., 2010). qPCR analysis of the samples in the “highly infected” category revealed high virus genome copies in *G. m. morsitans* (Figure 1B). When dissected however, none of these F_1_ progenies from GpSGHV-infected *G. m. morsitans* mothers showed any SGH symptoms. This is unlike in the *G. pallidipes* F_1_ progenies, which revealed 100% prevalence of SGH symptoms (data not shown), a result which was in agreement with our recent report (Boucias et al., 2013). Notably, the GpSGHV density levels in F_1_ progenies of *G. pallidipes* were significantly higher (*P* = 0.0014) compared to the virus-infected *G. m. morsitans* F_1_ progenies (Figure 1C). Importantly, the virus density levels in both the mock- and the virus-infected *G. m. morsitans* F_1_ progenies were comparable to those of the mock-infected *G. pallidipes* (see Figure 1C).

### Determination and characterization of *Glossina* SG proteomes

We then performed mass spectrometry on the SG protein extracts from the F_1_ progenies of *G. m. morsitans* flies with high viral titters and the *G. pallidipes* flies with overt SGH symptoms. Analyses of the GpSGHV-infected *G. m. morsitans* and *G. pallidipes* proteomes compared to their mock-infected counterparts resulted in 3815 unique peptides that mapped to 863 non-redundant proteins. Of these proteins, 87.7% (*n* = 757) were host (*Glossina*)-specific, while 8.5% (*n* = 73) and 3.8% (*n* = 32) were from GpSGHV and bacterial endosymbionts (*Wigglesworthia glossinidia* and *Sodalis glossinidius*), respectively. We then filtered out proteins with single and modified peptides, which resulted in 606 proteins, of which 540, 58, and 9 proteins were from the host, GpSGHV and *W. glossinidia*, respectively. All the identified proteins are detailed in Supplementary Material (Tables S1–S8).

#### Effects of GpSGHV infections on the overall SG protein expression patterns

**Figure 2:**
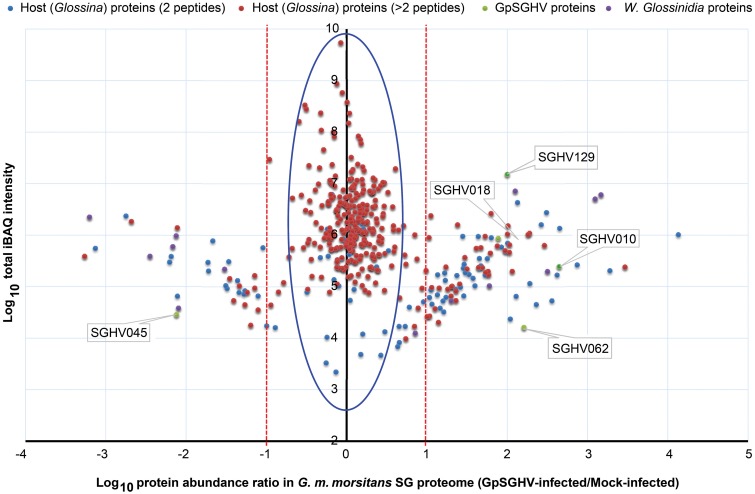
**Abundance distribution ratios of *G. m. morsitans* SG proteins**. The figure depicts the distribution of proteins detected in the SG proteome of *G. m. morsitans* infected by GpSGHV compared to the mock-infected controls. Shown are the host proteins that were detected by two (light blue) or more (red) peptides per protein. GpSGHV and *Wigglesworthia glossinidia* proteins are shown in green and purple, respectively. The proteins that were up-regulated and down-regulated in GpSGHV-infected SG are shown on the right and left sides of the Y-axis, respectively (in the blue large circle). The dotted red lines represent 10-fold protein regulation. iBAQ denotes intensity-based absolute quantification.

**Figure 3:**
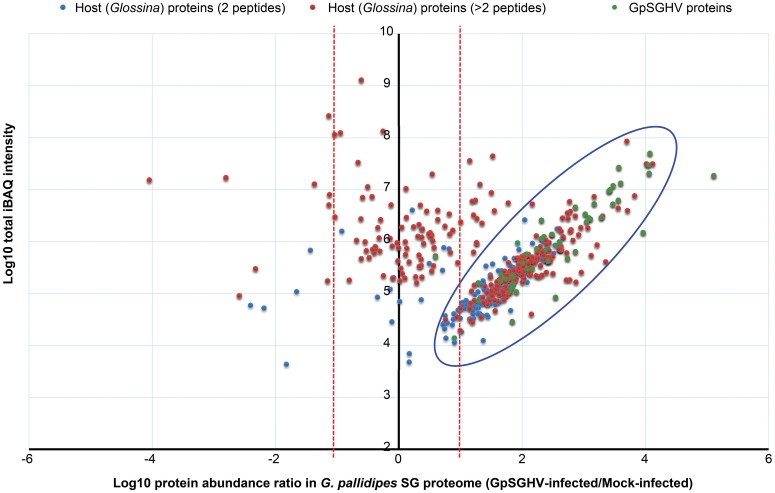
**Abundance distribution ratios of *G. pallidipes* SG proteins**. The figure depicts the distribution of proteins detected in the SG proteome of *G. pallidipes* infected by GpSGHV compared to the mock-infected controls. The host proteins detected by two or more peptides per protein are shown in blue and red, respectively, while the GpSGHV proteins are shown in green. The proteins that were up-regulated and down-regulated in GpSGHV-infected SG are shown on the right and left sides of the Y-axis, respectively. The large blue circle depicts proteins that were detectable in the SG proteome of GpSGHV-infected but not in the proteome of mock-infected *G. pallidipes*. Proteins which were not significantly modulated are depicted along the y-axis. The dotted red lines represent 10-fold protein regulation. iBAQ denotes intensity-based absolute quantification.

When we compared the LC-MS/MS data sets obtained from the GpSGHV-infected to mock-infected SGs, we found clear differential protein expression patterns in response to the virus infections in *G. pallidipes* and *G. m. morsitans* vs. their respective mock-infected controls (compare Figure 2 and Figure 3). From these two figures, GpSGHV infection had more drastic effects in the protein expression in the SGs of *G. pallidipes* than that of the *G. m. morsitans*. In the Figure 2, GpSGHV infection in *G. m. morsitans* had little overall effects on the host's SG protein expression patterns, i.e., majority of the proteins (confidently identified by ≥2 unique peptides per protein) were aligned around the y-axis (circled). On the other hand, a cohort of SG proteins were detectable in the proteome of GpSGHV infected *G. pallidipes*, but were hardly detectable in the proteome of the mock-infected flies (see circled proteins in Figure 3). Notably, in contrast to the *G. m. morsitans* SG proteins (Figure 2), only few proteins were not significantly affected by GpSGHV-infection in *G. pallidipes* (see proteins along the y-axis in Figure 3). Overall, comparing the GpSGHV-infected flies to their mock-infected counterparts, the majority of the host's SG proteins in *G. m. morsitans* had less than 10-fold up- or down-regulation compared to the proteome of *G. pallidipes* (see dotted red lines in Figures 2, 3).

#### Identification of differentially expressed proteins in response to GpSGHV infection

**Figure 4:**
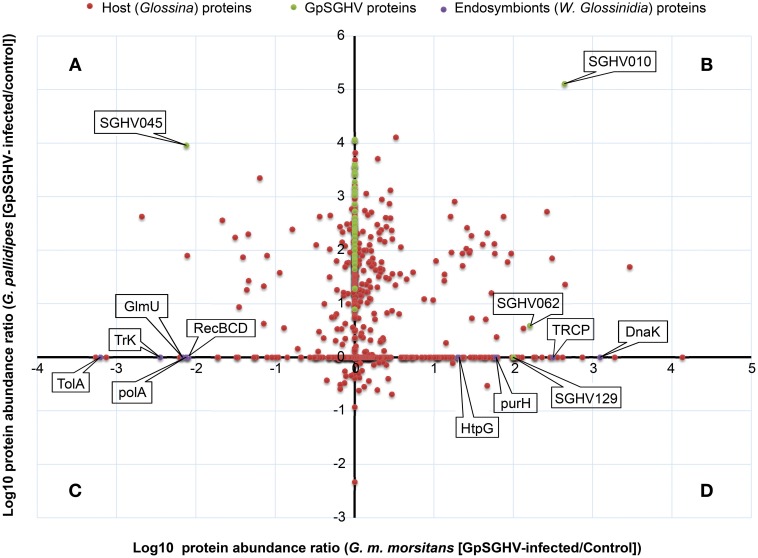
**Abundance distribution ratios of GpSGHV-infected *G. m. morsitans* and *G. pallidipes* SG proteins**. The figure shows a log-log PLOT of the host, viral and endosymbiont proteins (shown in red, green and purple, respectively). **(A)** Proteins down-regulated in *G. m. morsitans* but up-regulated in *G. pallidipes*. **(B)** Proteins up-regulated in both *G. m. morsitans* and *G. pallidipes*. **(C)** Proteins down-regulated in *G. pallidipes* but up-regulated in *G. m. morsitans*. **(D)** Proteins down-regulated in both *G. m. morsitans* and *G. pallidipes*. Proteins aligned along the Y-axis were detectable in *G. pallidipes* but were not detectable in *G. m. morsitans*, while the proteins aligned along the X-axis were detectable in *G. m. morsitans* but not detectable in *G. pallidipes.*

We then made a more comprehensive comparison of the GpSGHV-induced protein modulation by generating a log-log plot of the abundance distribution ratios of *G. m. morsitans* and *G. pallidipes* SG proteins. By combining the proteomics datasets obtained from the *G. m. morsitans* and *G. pallidipes* SG proteomes (Figures 2, 3, respectively), the identified host proteins fell into two broad categories. The first category consisted of proteins that were down-regulated or up-regulated in either or both *G. m. morsitans* and *G. pallidipes* proteomes. The second category consisted of the proteins that were detectable in one of the two *Glossina* species and in not the other. These categories are presented in the Figure 4.

Proteins in the first category consisted of four groups: First, compared to mock-infected controls, a total of 57 proteins were up-regulated in the GpSGHV-infected *G. pallidipes* SG proteome but were down-regulated in *G. m. morsitans* (Figure 4A; Table S1). Of the 57 proteins, 23 showed more than 100-fold up-regulation in *G. pallidipes* proteome and they were all down-regulated in virus-infected *G. m. morsitans* (Table 1). Second, 134 proteins were up-regulated in both GpSGHV-infected *G. m. morsitans* and *G. pallidipes* SG proteomes compared to their mock-infected counterparts (Figure 4B; Table S2). Third, compared to the mock-infected controls, 18 proteins were down-regulated in the virus-infected *G. pallidipes* but up-regulated *G. m. morsitans* (Figure 4C; Table S3), nine of which were up-regulated ≥ 5-fold in *G. m. morsitans* compared to the *G. pallidipes* SG proteomes (Table 2). Lastly, nine proteins were down-regulated in both virus-infected *G. m. morsitans* and *G. pallidipes* as measured from their proteomes (Figure 4D; Table S4).

**Table 1:** **Twenty-three host proteins that were more than 100-fold up-regulated in the GpSGHV-infected *G. pallidipes* but were down-regulated in the proteome of virus-infected *G. m. morsitans***.

**UniProt IDs**	**Protein names**	**Length [aa]**	**Mol. weight [kDa]**	**Functional characteristics/Annotation (Roles during virus infection)**	**References**
D3TP07	Proteasome subunit alpha-4 type	248	27.918	ATP/ubiquitin-dependent non-lysosomal proteolytic pathway; (host-virus interaction; blocking of protease activity and stimulates transcription trans-activation by viruses)	Krüger et al., 2001
D3TS03	ER glucose-regulated protein; (Hsp90)	716	81.953	Molecular chaperone; promote maturation, structural maintenance and regulation of proper folding of proteins involved in signal transduction; (virus-controlled transcriptional/translational switches)	Kariithi et al., 2011
D3TSB7	Ubiquitin/SUMO (small ubiquitin-related modifier) activating enzyme uBA1	567	64.293	Alters protein function, location, trafficking, or targeting to 26S proteasome for degradation; (SUMO is a partner protein to viral replication centers/virus assembly; the proteins sumoylate and therefore prompt viral gene expression, hence benefit viral replication)	Lallemand-Breitenbach and de Thé, 2010; Chen et al., 2013
D3TMU2	Integrin-linked kinase (ILK)	448	50.871	Diverse signaling pathways; ILKs are up-regulated in unregulated cell proliferation, migration, and inhibition of apoptotic arrest; (receptor-mediated viral entry and egress)	Edwards et al., 2004; Esfandiarei et al., 2006; Grove and Marsh, 2011
D3TM51	Mitochondrial oxoglutarate/malate carrier protein (OGC)	318	35.072	Mitochondria carrier (MC) protein family; (OGCs are up-regulated during virus infection as adaptive response to prevent mitochondrial injury)	Ripoli et al., 2010; Ohta and Nishiyama, 2011
D3TS86	40S ribosomal protein S16	141	15.952	Protein synthesis; Constituent proteins of stress granules (SGs) and processing bodies (P-bodies) that are involved in mRNA turnover (viruses modulate SGs and P-bodies to promote synthesis of viral proteins)	Lloyd, 2013; Reineke and Lloyd, 2013
D3TQA9	Ribosomal protein L19	204	24.083		
D3TQT3	Ribosomal protein L5	297	33.983		
D3TLP1	60s ribosomal protein L7	255	29.731		
D3TM09	Transketolase protein (TKTL) 2-like	627	68.103	Provide a link between the glycolytic, pentose-phosphate and nucleotide synthesis pathways; (during virus infections when rapid DNA synthesis is required, glucose carbon molecules are channeled toward nucleotides synthesis through TKTL pathway)	Chen et al., 2011; Noch and Khalili, 2012; Brault et al., 2013
D3TSC0	Hemomucin	549	60.823	(Salivary gland mucins are up-regulated during virus infection and therefore they represent insect host defense response to arbovirus infection)	Bishop-Lilly et al., 2010
D3TRZ3	E3 - ubiquitin ligase	150	16.537	Zinc ion-binding protein with specialized functions; (during virus infection, the protein targets specific cellular proteins for destruction by the ubiquitin proteasome system (UPS); viruses hijack UPS to promote favorable cellular environment for replication, or to block activation of host's defense mechanisms)	Eldin et al., 2009; Alcaide-Loridan and Jupin, 2012; Verchot, 2014
D3TRS6	Annexin	324	35.941	(Implicated in virus assembly on lipid rafts and directing virions to the cellular exocytotic machinery, thus aiding in non-lytic virus egress from infected cells)	Beaton et al., 2002; Harrist et al., 2009; Saxena et al., 2012
D3TME1	Annexin	319	35.299		
D3TMI0	Protein ZASP (z band alternatively-spliced PDZ-motif protein)	302	33.498	PDZ domains are found in cytoplasmic and adapter proteins involved in diverse cellular processes of significance to virus infection; (viruses modulate PDZ proteins to enhance their replication, dissemination in the host and transmission)	Golebiewski et al., 2011; Javier and Rice, 2011
D3TMN6	Eukaryotic translation initiation factor 3 subunit M (eIF3m)	387	44.081	eIF3m plays critical roles in promoting the initial translation of viral immediate early protein; (inhibition of eIF3m blocks virus infection)	Cheshenko et al., 2010
D3TNJ0	26S proteasome regulatory complex subunit RPN2/PSMD1	1005	111.24	Protein synthesis; enzyme regulatory activity; (the ubiquitin/26S proteasome system (UPS) is part of the unfolded protein response (UPR) machinery, an early event essential for persistent virus infection that benefits virus replication)	Verchot, 2014
D3TMA0	GTPase Rab2	213	23.568	Ras-like small GTPases are ‘molecular switches and key regulators of (vesicular) membrane traffic; (Rab GTPases regulate anterograde traffic between the ER, Golgi complex and cellular membranes)	Zenner et al., 2011
D3TLN8	Protein phosphatase 2A (PP2A)−29B	591	65.501	Hippo signaling pathway; (Viruses target specific PP2A enzymes to deregulate cellular pathways to counteract host antiviral defenses and promote viral progeny production)	Sontag, 2001; Guergnon et al., 2011
D3TPG7	G protein β-subunit-like protein	318	35.485	A WD-40 repeat containing protein implicated in signal transduction and transcription regulation to cell cycle control, cellular proliferation and apoptosis; (viruses highjack G-protein mediated signaling to drastically facilitate their infection and transmission)	Kirshner et al., 1999; Sodhi et al., 2004; Lin et al., 2005
D3TN12	Serine-arginine rich protein 55 (SRp55)	351	40.1	Conserved family of pre-mRNA splicing regulators; (viruses hijack SRps to increase production of virus progeny)	Fukuhara et al., 2006; Dubois et al., 2014
D3TP27	Hypothetical conserved (TcP-1-like) protein	174	20.525	Potentially involved in skeletal muscle myosin thick filament assembly	–
D3TMK9	Tailless-complex polypeptide-1 (TcP-1) zeta subunit	531	58.183	A chaperonin involved in the assembly of viral capsid	Lingappa et al., 1994; Inoue et al., 2011

Where applicable, the pathways in which the listed proteins are involved, and relevant supportive literature on their roles during the infection of the viruses in their hosts are indicated in columns 5 and 6, respectively.

**Table 2:** **Nine proteins that were more than 5-fold down-regulated in the GpSGHV-infected *G. pallidipes* but were up-regulated in the proteome of virus-infected *G. m. morsitans***.

**UniProt IDs**	**Protein names**	**Length [aa]**	**Mol. weight [kDa]**	**Functional characteristics/Annotation (Roles during virus infection)**	**References**
D3TRX7	Hypothetical conserved protein	161	18.487	Protein anoxia up-regulated 1-like, isoform X2 (*Musca domestica*); Prior to viral replication, the protein is up-regulated due to induction of ROS	Mutuel et al., 2010
D3TN39	26S proteasome non-ATPase regulatory subunit 3 (ATPase 3)	409	46.377	Regulates degradation of ubiquitinated proteins; down-regulated during viral infection	Lee et al., 2013
D3TLI6	Vacuolar (H^+^)-ATPase)–A	488	54.083	ATP-driven proton pump responsible for acidification of intracellular compartments such as endosomes; Involved in antiviral defense in silkworms; V-ATPase acidifies endosomes and/or lysosomes to make them competent to eradicate viruses; over-expression of V-ATPase significantly inhibits virus proliferation	Jefferies et al., 2008; Lu et al., 2013
D3TSC7	Vacuolar (H^+^)-ATPase)–B	614	68.093		
D3TLR6	Vacuolar (H^+^)-ATPase)–D	246	27.602		
D3TLB1	Vacuolar (H^+^)-ATPase)–E	226	26.002		
D3TR42	Mitochondrial ATP synthase (α-subunit)	552	59.358	Plays important roles in antiviral (e.g., WSSV) in shrimps; Involved in clearing of viruses through phagocytic engulfment	Wang et al., 2006; Badillo-Vargas et al., 2012
D3TR98	Mitochondrial Cytochrome bc_1_ complex (Rieske sub-unit)	258	27.577	Over-expression of Rieske subunit leads to increased oxidative metabolism as an adaptive response to pathogen infection in insects	Marie et al., 2014
D3TRB1	Mitochondrial processing peptidase (β-subunit)	454	50.391		

Where applicable, the pathways in which the listed proteins are involved, and relevant supportive literature on their roles during the infection of the viruses in their hosts are indicated in columns 5 and 6, respectively.

Similarly, the proteins that were detectable in the proteome of one of the two GpSGHV-infected tsetse species and not in the other fell into two main groups. First, compared to mock-infected flies, 189 proteins were detectable in the virus-infected *G. m. morsitans* SG proteome but not in that of *G. pallidipes* (Figure 4, *x*-axis), 65.1% (*n* = 123) of which were up-regulated (Table S5). Second, 133 proteins were detectable in *G. pallidipes* but not in *G. m. morsitans* (Figure 4, *y*-axis), 96.9% of which were up-regulated (Table S6).

A closer look at the proteomics data indicate that majority of the heavily modulated proteins in the SG proteome of *G. pallidipes* appear to be spread over a wide range of host pathways. These pathways included, among others: ATP/ubiquitin-dependent and 26S proteasome (UPS) pathways, integrin-liked kinase pathway, transketolase pathway, hippo signaling pathway and diverse signaling pathways (Table 1). In the case of *G. m. morsitans*, proteins potentially involved in the host's antiviral defenses appear to be quite dominant. Some of the antiviral defense-related systems included induction of innate immune response via reactive oxygen species (virus degradation), the ubiquitin/26S proteasome system, V-ATPase system (virus degradation via acidification of endosomes and or/lysosomes), the phagocytic engulfment system (to clear virus infection) and adaptive mitochondrial-mediated immune responses (interfere with production of progeny virus) (Table 2).

#### Detection of virus-/endosymbiont-specific proteins in *Glossina* SG proteomes

Whereas only five GpSGHV proteins (encoded by ORFs SGHV018, SGHV010, SGHV045, SGHV062 and SGHV129) were detectable in *G. m. morsitans*, a total of 58 proteins were detected in the SG proteome of *G. pallidipes* (See Figures 2–4; Table S7). These proteomics results reflects the findings we obtained from the qPCR assays described above. It should be noted that SGHV010 is the most abundant of all GpSGHV proteins; SGHV062 is a high-molecular weight (512 kDa) viral protein, while SGHV045 is a one of the major viral envelop proteins (Kariithi et al., 2013a). The high abundance and large size, respectively, of these virion proteins possibly explains their detection in *G. m. morsitans*. Further, proteins encoded by the ORFs SGHV018 and SGHV129 have not been detected in our previous proteomic studies in *G. pallidipes*, and were in the current study only detectable in the *G. m. morsitans* proteome but not in the *G. pallidipes* proteome (Figure 2). The failure to detect other viral proteins in *G. m. morsitans* does not necessarily imply complete absence of these proteins. Rather, their abundances could have been too low or their detection could have been masked by the highly abundant host proteins.

All the nine *Wigglesworthia* proteins were in the detected in the SG proteome of *G. m. morsitans* (Table S8), probably because unlike the *G. m. morsitans*, the genome of *G. pallidipes* is not yet available. Whereas four of the nine *Wigglesworthia* proteins were up-regulated in the virus-infected compared to mock-infected flies, five were down-regulated (Figure 4). The up-regulated proteins included transcription repair coupling factor, chaperone protein DnaK, chaperone protein HtpG, and IMP-cyclohydrolase. Down-regulated proteins included protein TolA, DNA polymerase I, exonuclease V, bifunctional enzyme GlmU and a potassium transporter protein. Since *Wigglesworthia* is housed within the host's bacteriocytes (Pais et al., 2008), the detection of *Wigglesworthia* proteins in the *G. m. morsitans* SGs suggest that these proteins likely “leak” into the hemolymph, potentially during bacteriocytes turnover, and eventually move from the hemocoel to the SGs.

## Discussion

### *G. m. morsitans* is less permissive to GpSGHV infections than *G. pallidipes*

Hytrosaviruses replicate primarily in the SG tissue of their insects hosts (Garcia-Maruniak et al., 2009). As such, one would expect that the virus-induced modulation of the SG microenvironment (i.e., morphological and biochemical/functional features of the tissue) results in the expression of various proteins/peptides specifically to the advantage of viral replication and dissemination. Whereas GpSGHV in *G. pallidipes* occurs in both asymptomatic and symptomatic infection states, the virus infection in other *Glossina* species is almost always asymptomatic. We have previously demonstrated the dynamics of the development of virus-induced SGH symptoms (Abd-Alla et al., 2010, 2013) and trans-generational transmission of the virus in *G. pallidipes* (Boucias et al., 2013). However, the case of GpSGHV infection in other *Glossina* species has not been investigated. Importantly, we have so far not observed any overt SGH symptoms in the *G. morsitans* colony maintained at the IPCL Seibersdorf, which was used as the source of the experimental flies we have described here.

In the current study, the effects of GpSGHV infections in *G. m. morsitans* were remarkably different from previous studies. For instance, virus injection into newly larvipositioned third-instar larvae of two Morsitans groups resulted in varying prevalence of SGH-like symptoms in developed adults, which ranged from 1.1% (Jura et al., 1993) to 4.0% (Kokwaro et al., 1990) in *G. m. morsitans*, and up to 100% in *G. m. centralis* (Sang et al., 1997). In our case, we did not observe any SGH symptoms in *G. m. morsitans*, potentially because whereas we injected the virus into newly eclosed adults, the researchers in the previous studies injected the virus suspensions into larvae. We therefore conclude that the injected virus is capable of infecting and replicating during ontogeny on the SGs during pupation (as evidenced from the previous studies). Further, the injected virus appears incapable of infecting and inducing overt SGH symptoms in fully differentiated SG cells in adults (as evidenced from our study).

Notably, the observed high virus titters in *G. m. morsitans* could represent DNA replication but may not represent production of infectious viral particles. The comparable virus titters between the virus-infected *G. m. morsitans* and the mock-infected *G. pallidipes* suggest that the virus may be undergoing only partial replication in adult cells to maintain steady-state titters throughout the adulthood. This is in agreement with previous studies in *G. pallidipes* whereby, utilizing a diagnostic PCR, Abd-Alla et al. (2007) detected GpSGHV PCR positives in 100% of colonized *G. pallidipes* that did not exhibit overt SGH symptoms. Taken together, the analyses of GpSGHV loads (by agarose gels), virus density levels (by qPCR), and protein expression (by LC-MS/MS) imply that either *G. m. morsitans*, at least in the SGs, is far less permissive to virus replication or that the virus undergoes limited replication in *G. m. morsitans* (whereby only a subset of genes are expressed) compared to *G. pallidipes*. Potentially, unlike in *G. pallidipes* where SGH symptoms can be overt, we are of the opinion that GpSGHV infection is entirely latent in *G. m. morsitans* as previously proposed by Kariithi et al. (2013b). Potentially, the difference in GpSGHV replication, i.e., partial replication or latency in *G. m. morsitans* vs. active replication in *G. pallidipes*, explains the differences in the repertoire of proteins detected in the SG proteomes of the two *Glossina* species.

### GpSGHV potentially modulates *G. pallidipes* key pathways for efficient infection

The clear GpSGHV-induced differential modulation of SG protein expression in *Glossina* raises the question of what host pathways are potentially globally regulated to facilitate successful virus infection. It is well known that for cellular entry and induction of pathogenesis, many viruses manipulate key host signaling pathways that globally regulate many cellular processes (Diehl and Schaal, 2013). So far, we have not been able to elucidate the precise mechanism(s) through which GpSGHV induces overt SGH symptoms, mainly because of a lack of cell culture system to support the virus multiplication (Arif and Pavlik, 2013). Therefore, the precise mechanisms of GpSGHV infection (cellular attachment, entry, intracellular trafficking, replication, maturation, and egress) in *Glossina* remains elusive. In an attempt to unravel the pathobiology of GpSGHV, we draw inferences from other virus-host systems that have been studied so far.

Of the proteins we identified in this study, proteins that showed significant differential expression patterns in virus-infected flies are particularly interesting since they potentially reflect involvement in GpSGHV pathogenesis. In this regard, the proteins that were significantly up-regulated in the SG proteome of *G. pallidipes* but down-regulated in that of *G. morsitans* (Table 1) are interesting to focus on. Also important were the nine proteins found to be up-regulated in proteome of *G. morsitans* but down-regulated in the proteome of *G. pallidipes* (Table 2). Notably, our annotation of the nine proteins revealed that these proteins may be involved in pathways related to the host's antiviral responses to virus infection (See Table 2 and the references therein). In the following sections, we briefly discuss the potential roles of the proteins stipulated in Tables 1, 2 with regard to viral entry into host cells, intracellular trafficking, and evasion of host's immune response, replication/translation and cellular proliferation.

#### Viral entry and intracellular trafficking

For entry, some viruses attach to host cell receptors thus inducing conformational changes that cause fusion of the viral envelope with the host's plasma membrane (Thorley et al., 2010). This is followed by delivery of the viral nucleocapsids into the cellular cytoplasm and uncoating of the viral genome. Integrin-linked kinases (ILKs), which were up-regulated in *G. pallidipes* (D3TMU2; Table 1), have been implicated in viral cellular entry. For instance, Kaposi's sarcoma-associated herpesvirus (KSHV) envelop glycoprotein B (gB) hijacks ILKs to induce the FAK-Src-PI3K-RhoGTPase signaling pathway (Naranatt et al., 2003; Sharma-Walia et al., 2005). Similar to the KSHV gB envelope protein, the GpSGHV SGHV038 protein, which was detected in the current study (Table S1), contains an arginyl-glycyl-aspartic acid (RGD) motif that may interact with the host's ILKs. Pending experimental validations, GpSGHV potentially employs an entry mechanism similar to KSHV (Krishnan et al., 2005).

Following cellular entry, a critical phase in viral pathogenesis is intracellular trafficking of viral nucleocapsids, a process that requires intricate signaling. One of the key pathway components targeted by several viruses is the GTPase Rab2 protein. Notably, this protein was found to be up-regulated in *G. pallidipes* (D3TMA0; Table 1), unlike in *G. m. morsitans*. GTPases regulate membrane trafficking, particularly in the formation, motility and docking of vesicles (Zerial and McBride, 2001). Some viruses activate GTPase-mediated pathways to facilitate their intracellular trafficking (Chien et al., 2006). For instance, in the absence of Rab 1a/b, herpes simplex virus 1 (HSV-1) was unable to traffic from the ER to cytoplasmic viral assembly complexes, leading to a build-up of un-enveloped viral particles in the cell cytoplasm (Zenner et al., 2011). GTPases were indeed found to be up-regulated in shrimps infected with whispovirus (Wu and Zhang, 2007), another large invertebrate dsDNA virus like GpSGHV. It is tempting to postulate that GpSGHV up-regulates GTPases for intracellular trafficking in *G. pallidipes*, especially because the virus genome shares at least 28 putative homologs with the above-mentioned large dsDNA viruses, including HSV and whispoviruses (Abd-Alla et al., 2008).

#### Viral replication and dissemination in the host

Apart from the knowledge that the host's SG is the primary replication organ for GpSGHV (Garcia-Maruniak et al., 2009), and that the virus is transmitted from the infected mother to the progeny via the milk gland secretions (Boucias et al., 2013), the precise virus replication and dissemination mechanisms are unknown. By comparing our data with the data available from other virus-host systems, it is possible to postulate theories on GpSGHV replication and dissemination in *Glossina*.

Some viruses modulate the mitochondrial transport machinery to provide energy necessary for replication, especially for the viruses whose genomes are A+T-rich (Ohta and Nishiyama, 2011; Anand and Tikoo, 2013). The GpSGHV genome is A+T-rich (72%; Abd-Alla et al., 2008), implying that virus-modulation of *Glossina* mitochondrial transport machinery is a good possibility. The mitochondrial oxoglutarate/malate carrier (OGC) protein is important for the tricarboxylic acid cycle (TCA), gluconeogenesis and nitrogen metabolism (Cappello et al., 2006). OGC is reportedly up-regulated as an adaptive response to prevent mitochondrial injury (Ripoli et al., 2010). Thus, the up-regulation of OGC (D3TM51) in *G. pallidipes* (Table 1) may be GpSGHV-induced when robust virus replication occurs, especially in the event of overt SGH symptoms. Another host protein targeted by viruses to facilitate replication is the transketolase (TKTL)-2 (D3TM09). We found TKTL protein to be up-regulated more than 100-fold in *G. pallidipes* (See Table 1) unlike in *G. m. morsitans*. TKTL provides a link between the glycolytic, pentose-phosphate, and nucleotide synthesis pathways (Brault et al., 2013). During active virus replication when rapid DNA synthesis is required, carbohydrate molecules are channeled to the DNA synthesis machinery through the TKTL pathway, a process of utmost importance in proliferating tissues (Chen et al., 2011). This is of particular interest in this case of induction of the SGH symptoms in *G. pallidipes*, especially because SGH is mainly due to cell proliferation (Guerra et al., 2013). Since the TKTL pathway allows synthesis of ribose without the need of oxygen, GpSGHV may highjack the TKTL pathway to circumvent the need for oxygen (Noch and Khalili, 2012), thus allowing rapid GpSGHV genome replication.

Another host protein involved in viral replication is proteasome α-4 (D3TP07; Table 1), a key protein in the ATP/ubiquitin-dependent non-lysosomal proteolytic pathway. For instance, the interaction of proteasome α-subunit PSMA7 with hepatitis C virus (HCV) led to an inhibition of host protease activity and thus stimulated transcription trans-activation by HCV (Krüger et al., 2001). Several other host proteins involved in viral replication that were detected in the current study included eIF3m (D3TMN6; Cheshenko et al., 2010), molecular chaperones (e.g., hsp90; D3TS03; Kariithi et al., 2011) and 26S proteasome regulatory complex proteins (D3TNJ0; Verchot, 2014; See Table 1). The hypothetical conserved protein (D3TRX7; Table 2) is 100% identical to the *M. domestica* anoxia up-regulated 1-like protein and its expression in our case is virus-induced. Mutuel et al. (2010) reported a significant induction of reactive oxygen species (ROS) in the tracheal and fat body systems of lepidopteran insects early in infection with *Junonia coenia* densovirus. The authors made this observation prior to viral replication before any detectable disease symptoms. Interestingly, decrease of ROS induction positively correlated with exponential phase of viral infection. It has been proposed that GpSGHV may replicate in the host's fat bodies, and that the host's tracheal system provides a conduit for the virus transmission (Kariithi, 2013). It is likely that this and perhaps other similar proteins play roles during GpSGHV replication. Taken together, our data provide potential targets for future investigations of how GpSGHV replicates and is disseminated in the host.

#### Viral evasion of host's immune responses

Upon successful cellular entry, viruses must evade the host's immune responses, a process for which the host's ubiquitin/proteasome system (UPS) has significant roles. In the current study, we detected the main components of the UPS, i.e., E3 ligase (D3TNJ0) and 26S proteasome (D3TRZ3) (Table 1). The UPS is essential for persistent infection of some viruses. For instance, plant RNA viruses in the family *Luteoviridae* (genera *Poleroviruses* and *Enamoviruses*) encode viral suppressors of RNA silencing (VSRs) that hijack the UPS components to promote degradation of key components of the host's RNA-interference (RNAi) system, thereby promoting virus replication (Verchot, 2014). DNA viruses are known to be under host RNAi surveillance. These include invertebrate iridoviruses (Bronkhorst et al., 2012; Kemp et al., 2013), baculoviruses (Jayachandran et al., 2012), densoviruses (Ma et al., 2011), whispoviruses (Huang and Zhang, 2013), and plant viruses (Blevins et al., 2006). As stated above, GpSGHV infections are frequently observed, but remain asymptomatic and seldom result in SGH symptoms both in nature and in laboratory-bred *Glossina* species. Although the questions of how GpSGHV infection progresses from a covert asymptomatic infection to an overt symptomatic infection are yet to be answered, we speculate that the virus is under host RNAi surveillance, hence components of the host's UPS systems form ideal candidates for further studies.

Another group of host antiviral defense proteins detected in this study were the V-ATPases (D3TLI6; D3TSC7; D3TLR6; and D3TLB1; Table 2), whose activity leads to acidification of intracellular compartments, necessary for multiple cellular processes (Jefferies et al., 2008). Recently, Lu et al. (2013) reported that over-expression of V-ATPase in *Bombyx mori* nucleopolyhedrovirus (BmNPV)-infected cells significantly inhibited viral proliferation. Potentially, the acidification of endosomes and lysosomes by V-ATPase renders these organelles competent for viral degradation. It is therefore not surprising that in the current study, V-ATPase were up-regulated in the SG proteome of *G. m. morsitans* as opposed to that of *G. pallidipes* (Table 2) as the former appear to be less permissive to GpSGHV replication compared to the latter. Similar to the V-ATPase, mitochondrial ATP synthase was down-regulated during white spot syndrome virus (WSSV) infection in shrimps (Wang et al., 2006).

The ubiquinol-cytochrome c reductase iron-sulfur subunit (Rieske subunit/bc_1_) detected in the current study (D3TR98; Table 2) was demonstrated to be up-regulated during the infection of *Anopheles gambiae* by *Plasmodium falciparum* (Marie et al., 2014). The up-regulation of bc_1_ in these two cases could have been due to the presence of the parasites in the mosquito, which could be a response involved in parasite resistance. The α- and β-subunits of mitochondrial processing peptidase (MPP; D3TRB1; Table 2) are homologous to the core 2 and core 1 proteins of the bc1 complex (Braun and Schmitz, 1995). MPP and bc_1_ complex appear to have similar pathogen-induced modulation patterns, and their up-regulation in *G. m. morsitans* may be an adaptive antiviral host resistant response.

#### Viral persistent infection and induction of cellular pathology

It is still not clear how GpSGHV induces cellular proliferation in the host's SG tissue. However, other studies have demonstrated that some viruses induce cellular proliferation via modulation of specific signaling pathways. Protein phosphatase 2A (PP2A; D3TLN8; Table 1) is critical in the regulation of cell proliferation, signal transduction, cytoskeletal dynamics, and apoptosis (Seshacharyulu et al., 2013). Some viral proteins such as the small T antigen of SV40 specifically target and directly interact with and displace PP2A's scaffolding B subunit thereby inducing cellular proliferation (Guergnon et al., 2011). To activate intracellular signaling pathways, some viruses use various approaches to hijack the G-protein-coupled receptors (GPCRs; D3TPG7; Table 1), leading to enhancement of viral pathogenesis (Sodhi et al., 2004; Lin et al., 2005). Other viruses such as KSHV encode potent and constitutively active GPCR homologs that modulate cellular proliferation (Kirshner et al., 1999). Hypothetically, some GpSGHV envelop proteins (Table S1) could interact with host proteins to trigger signaling pathways resulting in hyperplasia as has been reported in other viruses such as the fowl poxvirus (Afonso et al., 2000). It is interesting to experimentally validate whether these virus and/or host proteins are actually involved in the development of SGH in *Glossina*.

#### Viral assembly and induction of SGH symptoms

An important step during assembly of viruses is processing of viral mRNAs, which in some cases involve the host *trans*-acting splicing factors such as serine/arginine-rich proteins (SRps; Akopian et al., 2013). To ensure production of their own protein diversity, adenoviruses, HSV-1, influenza A viruses (IAV) and HIV manipulate mRNA splicing by phosphorylating SRps (Estmer-Nilsson et al., 2001; Sciabica et al., 2003; Fukuhara et al., 2006; Dubois et al., 2014). Therefore, it not surprising that in the current study, SRp 55 (D3TN12) was up-regulated in GpSGHV-infected *G. pallidipes* (active viral replication), but down-regulated in *G. m. morsitans* (less permissive to viral replication) (Table 1). However, it is currently unknown how GpSGHV mRNAs are processed. Another host protein involved in viral assembly is tailless-complex polypeptide protein-1 (TcP-1; D3TP27 and D3TMK9; Table 1). For instance, TcP-1 has been implicated in the assembly of hepatitis B/C virus capsids (Lingappa et al., 1994; Inoue et al., 2011), while annexins (D3TRS6 and D3TME1; Table 1) are involved in the HIV-1 assembly in lipid rafts (Harrist et al., 2009; Saxena et al., 2012). Other proteins that may be involved in viral assembly include the 26S proteasome non-ATPase regulatory subunit 3 (ATPase 3; D3TN39), which was down-regulated as seen in the SG proteome of *G. pallidipes* (Table 2). Potentially, this protein may regulate (by blocking) degradation of viral proteins. A gene similar to ATPase 3 was found to be down-regulated more than 10-fold in the rice stripe virus (RSV) infected small brown plant hopper, *Laodelphax striatellus* (Lee et al., 2013).

Induction of the SGH symptoms is possibly a reflection of active production of viable progeny virus particles. During active virus progeny production, enveloped viruses are known to depend on the endoplasmic reticulum (ER) for maturation of viral envelope glycoproteins, and proteins involved in the formation of replication complexes, assembly, envelopment and genome packaging (Medigeshi et al., 2007; Scheel and Rice, 2013). This imposes a tremendous protein load in the ER, leading to ER stress. Consequently, ER stress results in the induction of the unfolded protein response (UPR), an evolutionary conserved prosurvival pathway that signals the nucleus to induce the expression of various chaperones (Walter and Ron, 2011). In some cases, interaction between the induced chaperones and viral proteins is critical for processing of viral proteins and assembly of mature virions. During prolonged and overwhelming ER stress, UPR switches from being prosurvival to proaptotic (Szegezdi et al., 2006). Prolonged virus-induced ER stress/UPR responses modulate a variety of signaling pathways that contribute to viral pathogenesis (Fung and Liu, 2014), and may lead to cellular proliferation and hypertrophy. The up-regulation of UPR-associated proteins and several molecular chaperones in flies with SGH symptoms (see Table 1) implicates the UPR/ER stress machinery in the development of overt SGH symptoms in *G. pallidipes*. As discussed above, the TKTL pathway may also be involved in the expression of overt SGH symptoms in *G. pallidipes*. The current data provide potential targets for development of rationally designed antiviral strategies in large tsetse fly rearings for sterile insect technique.

## Conclusions

The data presented in this study provide hints as to why *G. m. morsitans* is much less susceptible host to GpSGHV infection compared to *G. pallidipes*. The known and/or putative functions inferred from sequence similarity analyses revealed that the differentially modulated proteins we have identified are potentially involved in various aspects of GpSGHV pathogenesis. Specifically, and like in many other viruses, GpSGHV appears to deploy a repertoire of strategies to exploit the host intracellular signaling pathways for replication, especially in *G. pallidipes*. In the case of *G. m. morsitans*, host proteins involved in antiviral defense systems appeared to be dominant. Some of the pathways that appear to be targets of the virus include the UPR and TKTL pathways, implicating their involvement in expression of overt SGH symptoms in *G. pallidipes*. The proteins involved in these pathways deserve further functional (experimental) validations to understand the relevance of the differences in their expression patterns. The current study is a critical baseline data in a new Coordinated Research Project (CRP) initiated by IAEA, aimed at gaining a deeper knowledge of the *Glossina*/symbiont/GpSGHV tripartite interactions and how these interactions affect *Trypanosoma* parasite transmission (Van Den Abbeele et al., 2013). We have designed RNAi bioassays to further investigate how asymptomatic GpSGHV infection is maintained in *Glossina*. Candidate proteins experimentally validated as essential for efficient GpSGHV pathogenesis are ideal targets for developing rationally designed antiviral strategies to control the virus infections in tsetse mass rearing facilities. In a larger view, our data are important for future studies on molecular and biochemical routes employed by members of the new entrants into the family of insect viruses, the *Hytrosaviridae*.

## Author contributions

HK, İİ, JV, MV, and AA participated in the design of the study. AA and IM set up the bioassays. HK and İİ processed and quantified the salivary gland proteins. SB performed the LC-MS/MS measurements. SB and HK analyzed the proteomics data sets. HK and EM annotated/characterized the proteins. HK wrote the manuscript. JV, MV, AA, İİ, EM, EO, and SN contributed in writing the paper, providing critical comments and suggestions. All the authors read and approved the final manuscript.

### Conflict of interest statement

The authors declare that the research was conducted in the absence of any commercial or financial relationships that could be construed as a potential conflict of interest.
