# Supplementary material for: Comparative Analysis of Salivary Gland Proteomes of Two Glossina Species that Exhibit Differential Hytrosavirus Pathologies
Source: Front Microbiol. 2016 Feb 9;7:89. doi: 10.3389/fmicb.2016.00089 (PMC4746320; doi:10.3389/fmicb.2016.00089)
Supplement: Supplementary file 1 [file DataSheet1.docx]

Supplementary Material

Comparative analysis of salivary gland proteomes of two *Glossina* species with differential hytrosavirus pathologies

Henry M. Kariithi^*^, İkbal Agah İnce, Sjef Boeren, Edwin K. Murungi, Irene K. Meki, Everlyne A. Otieno, Steven R. G. Nyanjom, Monique M. van Oers, Just M. Vlak and Adly M. M. Abd-Alla^*^

*** Correspondence:** Adly A. M. M. Abd-Alla: a.m.m.abd-alla@iaea.org; Henry M. Kariithi: henry.kariithi@kalro.org

**Table S 1: Annotation of fifty-seven host protein that were up-regulated in the SG proteome of GpSGHV-infected *G. pallidipes* but were down-regulated in the proteome of *G. m. morsitans*:** The number of unique peptides that identified the proteins in *G. m. morsitans* and *G. pallidipes*, respectively, are shown in the square brackets in column 4. The amino acid coordinates of the signature domains in the protein sequences are indicated in the square brackets in column 6. The proteins listed in this table are also depicted in Figure 4, Panel A.

| **UniProt ID** | **Protein Name/Description** | **Mol. Weight [kDa]** | **Peptides [Unique]** | **Sequence coverage [%]** | **Predicted signature/conserved domains; [amino acid coordinates in the protein sequence]** |
| --- | --- | --- | --- | --- | --- |
| D3TP07 | Alpha proteasome subunit | 27.918 | 5 [4; 3] | 3.2 | Nucleophile aminohydrolases N-terminal domain [3-239] |
| D3TS03 | Endoplasmic reticulum glucose-regulated protein | 81.953 | 22 [17; 17] | 44.7 | Hsp90 [5-213], Ribosomal S5 domain [276-531] |
| D3TP87 | Elongation factor 2 | 94.415 | 30 [22; 13] | 3.3 | GTP binding [18-300]/V [725-830] domains |
| D3TQC5 | Muscle LIM protein at 84B | 52.909 | 15 [10; 8] | 9.4 | Zinc finger (LIM)-type domains [7-72; 113-179; 216-278; 318-388; 414-479] |
| D3TNC2 | Radixin | 67.922 | 19 [17; 5] | 8.9 | FERM domain [6-296] |
| D3TSB7 | uBA-1 | 64.293 | 6 [5; 4] | 20.1 | UBA/ThiF-type NAD/FAD binding domain [6-119] |
| D3TMU2 | Integrin-linked kinase | 50.871 | 4 [3; 3] | 12.9 | Ankyrin repeat domain [5-147], Protein kinase-like domain [173-443] |
| D3TM51 | Mitochondrial OGC carrier | 35.072 | 8 [2; 6] | 2.4 | Mitochondrial carrier domain [18-105; 112-206; 218-307] |
| D3TS86 | 40S ribosomal protein S16 | 15.952 | 6 [5; 5] | 17.8 | Ribosomal S9 [9-141] |
| D3TM01 | Adaptor protein enigma | 38.461 | 12 [12; 6] | 5.6 | LIM domain [39-91; 95-152; 156-213; 217-272; 276-335] |
| D3TQG6 | Dead box ATP-dependent RNA helicase | 45.629 | 21 [14; 13] | 9.2 | DEAD/DEAH box helicase domain [54-220], Helicase conserved C-terminal domain [287-364] |
| D3TM09 | Transketolase | 68.103 | 11 [4; 9] | 12.1 | Thiamine-[13-286]/Pyrimidine-binding [318-484]/C-terminal [495-617] domains |
| D3TMD9 | Heat shock protein β-1 | 20.119 | 6 [5; 3] | 24 | HSP20 [81-175] |
| Q694A5 | Putative thioredoxin peroxidase 1 | 21.755 | 9 [7; 6] | 21.3 | AhpC-TSA domains [4-137; 157-192] |
| D3TNR1 | Ribosomal protein L17 | 21.58 | 7 [3; 7] | 41.3 | Ribosomal L22 [17-152] |
| D3TSA6 | Electron transfer flavoprotein ubiquinone oxidoreductase | 66.394 | 7 [5; 2] | 28 | Electron transfer flavoprotein-ubiquinone oxidoreductase [451-559] |
| D3TSC0 | Hemomucin | 60.823 | 11 [11; 2] | 1.8 | Strictosidine synthase [173-259] |
| D3TLC7 | Isovaleryl-CoA dehydrogenase | 46.503 | 4 [3; 2] | 45.3 | N- [44-158], middle [162-214] and C-terminal [270-419] domains |
| D3TL47 | Putative cysteine proteinase TIN-ag-RP | 43.976 | 19 [17; 9] | 2.3 | Peptidase C1 [142-374] |
| D3TRZ3 | E3 ubiquitin ligase | 16.537 | 5 [4; 2] | 61.3 | Zinc knuckle [5-21; 40-57; 60-77; 81-98; 108-125; 128-145] |
| D3TS62 | Troponin | 47.19 | 25 [22; 11] | 8.1 | Troponin [147-246] |
| D3TQA9 | Ribosomal protein L19 | 24.083 | 5 [2; 3] | 27.4 | Ribosomal L19e [2-149] |
| D3TQM6 | Membrane trafficking protein | 25.125 | 5 [3; 5] | 14 | EMP24­GP25L [20-211] |
| D3TRS6 | Annexin | 35.941 | 8 [6; 5] | 6.2 | Annexin [25-90; 97-162; 180-246; 256-321] |
| D3TSD9 | Hydroxyacyl-CoA dehydrogenase | 83.429 | 15 [14; 9] | 9.2 | NAD binding [366-545]/C-terminal [547-642] domains |
| D3TL24 | 40S ribosomal protein S8 | 23.736 | 9 [5; 7] | 37.5 | Ribosomal S8e [1-191] |
| D3TMI0 | Hypothetical conserved protein | 33.498 | 10 [2; 2] | 5.2 | PDZ domain [9-88] |
| D3TRY7 | Crystallin alpha B | 21.335 | 9 [5; 5] | 14.1 | HSP20 [73-170] |
| D3TM93 | Putative cargo transport protein EMp24 | 23.545 | 4 [2; 4] | 43.8 | EMP24-GP25L [23-202] |
| D3TPX7 | Carboxyl-esterase | 65.444 | 3 [2; 2] | 15.7 | Co-esterase [11-556] |
| D3TNV8 | Elongation factor 1-alpha | 50.272 | 17 [16; 6] | 15.3 | Elongation factor Tu; GTP binding [5-238], domains 2 [260-327] and C-terminal domain [333-442] |
| D3TQT3 | Ribosomal protein L5 | 33.983 | 8 [5; 6] | 7.4 | Ribosomal L18; p [26-173] and C-terminal [192-283] domains |
| D3TLE7 | Thioredoxin reductase-1 | 54.124 | 7 [5; 4] | 32.6 | Pyridine nucleotide-disulphide oxidoreductase [18-340], dimerisation domain [370-483] |
| D3TNH4 | Nuclear envelope protein lamin | 70.547 | 28 [13; 27] | 15.5 | Lamin Tail Domain [460-577] |
| D3TNT0 | Thiol-disulfide isomerase | 46.74 | 8 [8; 3] | 28.5 | Thioredoxin domains [28-137; 65-351] |
| D3TLV4 | Rab protein 1 | 22.732 | 8 [8; 2] | 5.7 | Ras domain [13-174] |
| D3TLP1 | 60s ribosomal protein L7 | 29.731 | 6 [5; 3] | 10.1 | Ribosomal L30; N-terminal [23-93] and L7e [95-146] domains |
| D3TL23 | Mannose lectin ERGIC-53 | 54.86 | 10 [8; 6] | 9.7 | Lectin leg like domain[3-229] |
| D3TLL5 | Ribosomal protein L22 | 30.846 | 8 [6; 3] | 3.2 | Ribosomal L22e [174-285] |
| D3TLS8 | 4-hydroxybutyrate coenzyme A transferase | 52.059 | 7 [3; 4] | 29.3 | N- [48-221]/C-terminal [312-466] domains |
| D3TN16 | Synaptobrevin/VAMP-like protein | 24.433 | 6 [3; 5] | 4 | Longin [36-118], Synaptobrevin [129-211] |
| D3TME1 | Annexin | 35.299 | 5 [2; 2] | 36.1 | Annexin [19-84; 91-156; 170-240; 250-315] |
| D3TN36 | Repressor splicing factor 1 | 19.851 | 7 [2; 6] | 11.7 | RNA recognition motif [RRM] domain [9-74] |
| D3TSA8 | Ras-related protein Rab-7 | 23.508 | 6 [5; 2] | 28 | Ras [10-175] |
| D3TMN6 | Eukaryotic translation initiation factor 3 subunit M | 44.081 | 7 [2; 4] | 12.2 | PCI domain [236-337] |
| D3TLY7 | Putative serine protease | 81.952 | 6 [3; 3] | 44.5 | N-terminal beta-propeller [15-427]/Peptidase S9 [488-713] domains |
| D3TLP4 | 60s ribosomal protein L10 | 25.598 | 7 [3; 6] | 48.3 | Ribosomal L16 [5-167] |
| D3TNJ0 | 26S proteasome regulatory complex subunit RPN2/PSMD1 | 111.24 | 9 [3; 7] | 4.9 | Proteasome/cyclosome repeat [456-489; 491-525; 526-560], HEAT repeats [614-708] |
| Q0QHL0 | Mitochondria isocitrate dehydrogenase [NAD] subunit | 40.517 | 7 [3; 3] | 34.2 | Iso-DH [41-369] |
| D3TMA0 | GTPase Rab2 | 23.568 | 5 [2; 4] | 34.4 | Ras [8-169] |
| D3TM06 | Chaperonin complex component TcP-1 theta subunit CCT8 | 59.889 | 11 [2; 11] | 4.8 | cpn60-TCP-1 [39-536] |
| D3TLN8 | Phosphatase 2A at 29B | 65.501 | 8 [4; 6] | 4.2 | HEAT domains [15-115; 168-198; 207-237; 246-276] |
| D3TMH9 | Hypothetical conserved protein | 16.198 | 7 [1; 1] | 10.5 | - |
| D3TPG7 | G protein beta subunit-like protein | 35.485 | 8 [2; 5] | 16.7 | WD domain [6-44; 54-92; 96-134] |
| D3TN12 | Serine-arginine protein 55 | 40.1 | 2 [2; 2] | 22.6 | RNA recognition motif domains [6-68; 122-187] |
| D3TP27 | Hypothetical conserved protein | 20.525 | 14 [1; 1] | 14.3 | None |
| D3TMK9 | TcP-1 zeta subunit | 58.183 | 9 [6; 5] | 24.7 | Cpn60-TCP1 [30-525] |

HEAT repeats, *H*untingtin, elongation factor 3 (*E*F3), protein phosphatase 2A (PP2*A*), and the yeast kinase *T*OR1; Hsp, heat shock protein; TcP-1, Chaperonin complex component; LIM, Zinc-binding domain present in three proteins: Lin-11, Isl-1 and Mec-3; TIN-ag, tubulointerstitial nephritis antigen-related protein; ERGIC, endoplasmic reticulum (ER)-Golgi intermediate compartment; FERM domain, band Four-point-one/Ezrin/Radixin/Moesin domain; UBA/ThiF, ubiquitin activating enzyme/thiamine hydroxyethylthiazole F; AhpC-TSA, Alkyl hydro peroxide reductase subunit C/Thiol-specific antioxidant; PDZ domain, domain present in postsynaptic density (PSD)-95, Discs large (Dlg) and zonula occludens (ZO)-1/2 proteins; EMP24­GP25L, endosomal membrane protein-24_membrane glycoprotein-25L; PCI domain, proteasome, constitutive photomorphogenic-9 (COP9), initiator factor-3.

**Table S 2: Annotation of one hundred thirty-four proteins that were up-regulated in the SG proteomes of both GpSGHV-infected *G. m. morsitans* and *G. pallidipes***: The number of unique peptides that identified the proteins in *G. m. morsitans* and *G. pallidipes*, respectively, are shown in the square brackets in column 4. The amino acid coordinates of the signature domains in the protein sequences are indicated in the square brackets in column 6. The proteins listed in this table are also depicted in Figure 4, Panel B.

| **UniProt ID** | **Protein Name [Description]** | **Mol. Weight [kDa]** | **Peptides [Unique]** | **Sequence Coverage [%]** | **Predicted signature/conserved domains; [amino acid coordinates in the protein sequence]** |
| --- | --- | --- | --- | --- | --- |
| D3TNH8 | Ubiquitin carboxyl-terminal hydrolase | 25.796 | 5 [4; 2] | 9.8 | Peptidase C12 [3-211] |
| D3TLI4 | Eukaryotic initiation factor 5C | 48.734 | 5 [2; 3] | 9.7 | W2 [336-416] |
| D3TS02 | Hypothetical protein | 39.369 | 7 [2; 5] | 26 | Mo25-like [1-336] |
| D3TML7 | Asparaginyl-tRNA synthetase | 63.99 | 6 [2;5] | 22.7 | OB-fold nucleic acid binding [138-218]/class II [235-554] domains |
| D3TKL3 | Histone H3 | 15.257 | 4 [2; 3] | 11.1 | Core histone H2A/H2B/H3/H4 [58-132] |
| D3TQ27 | Actin depolymerizing factor | 17.036 | 4 [3; 3] | 4.1 | ADF-H domain [9-143] |
| D3TKJ4 | 60S ribosomal protein L31 | 14.331 | 5 [2; 4] | 10.2 | Ribosomal protein L31e [15-98] |
| D3TLM1 | Ribosomal protein L4 | 46.09 | 14 [9; 9] | 19.5 | Ribosomal protein L4/L1 [25-266]/ C-terminal [278-356] domains |
| D3TQU5 | 60s ribosomal protein L14 | 19.191 | 7 [6; 4] | 79.5 | Ribosomal L14 domains [46-122] |
| D3TLG1 | Aldo/keto reductase | 36.359 | 8 [4; 6] | 23.3 | Aldo-keto reductase domain [19-295] |
| Q2PZ04 | Lipophorin | 48.751 | 6 [4; 6] | 18 | Vitellogenin lipid transport domain [40-407] |
| D3TS11 | Aldehyde dehydrogenase | 59.013 | 8 [8; 2] | 10.9 | Aldedh [63-527] |
| D3TP17 | Ras-related GTPase | 23.118 | 2 [2; 2] | 18.5 | Ras [19-180] |
| D3TRT4 | Sideroflexin | 35.042 | 3 [2; 2] | 3.8 | MTC [14-320] |
| D3TP82 | Rab protein 6 | 23.304 | 5 [3; 3] | 2.4 | Ras [14-174] |
| D3TN37 | GTP-binding ADP-ribosylation factor | 20.34 | 3 [1; 1] | 11.6 | Arf [5-177] |
| D3TQ43 | Gamma-glutamyl phosphate reductase | 84.319 | 8 [7; 4] | 35.3 | Amino acid kinase [60-320], Alde-DH [336-656] |
| D3TNE7 | Signal peptidase I | 20.981 | 3 [2; 3] | 38.4 | Peptidase S24-like [57-135] |
| D3TMN9 | 60s ribosomal protein L23 | 29.487 | 2 [2; 2] | 6.6 | Ribosomal protein L23 [190-269] |
| D3TLE4 | Aspartyl tRNA synthetase | 59.946 | 6 [2; 5] | 7.2 | OB-fold nucleic acid binding [96-181]/2 [212-534] domains |
| D3TQA3 | Malic enzyme | 64.712 | 4 [3; 3] | 12.1 | Malic M domains [100-281; 291-544] |
| D3TRS5 | Vesicle coat complex COPII subunit SEC23 | 88.168 | 10 [3; 7] | 13.2 | Sec23/Sec24 zinc finger [57-98], Sec23/Sec24 trunk domain [126-406], Sec23/Sec24 beta-sandwich domain [417-520], Sec23 helical [535-635], Gelsolin [644-734] |
| D3TM41 | Eukaryotic translation initiation factor 3 subunit E | 51.122 | 5 [2; 4] | 35.3 | eIF3 subunit 6 N terminal domain [5-136], PCI domain [284-389] |
| D3TN28 | T-complex protein 1 subunit gamma | 59.361 | 15 [3; 14] | 5.2 | Cpn60-TCP1 [34-526] |
| D3TQX4 | 60S ribosomal protein L18a | 20.957 | 4 [2; 3] | 26.5 | Ribosomal L18ae/LX protein domain [7-130] |
| D3TLV7 | Chaperonin complex (TcP-1) eta subunit CCT7 | 59.161 | 7 [2; 6] | 29.1 | Cpn60-TCP1 [31-524] |
| D3TMM8 | Ras suppressor protein | 32.594 | 5 [3; 3] | 12.2 | LRR 8 [79-137]/LRR 1 [151-172]/LRR 4 [173-220] |
| D3TPI8 | Ribosomal protein L15 | 24.324 | 3 [2; 3] | 40.3 | Ribosomal L15e [4-195] |
| D3TRV3 | Seryl-tRNA synthetase | 56.035 | 4 [2; 3] | 9.2 | N-terminal [2-112]/class II core [204-383] domains |
| D3TQ47 | 3-hydroxyisobutyrate dehydrogenase | 34.382 | 6 [4; 4] | 44.8 | NAD binding domains [30-190; 192-319] |
| D3TMD6 | Fructose-1,6-bisphosphatase | 36.338 | 5 [3; 2] | 26.9 | FBPase [13-336] |
| D3TNF7 | Glutaryl-CoA dehydrogenase | 45.626 | 4 [3; 2] | 25.7 | N- [43-154]/middle domain [158-212]/C-terminal [266-413] domains |
| D3TP64 | Tropomyosin 1 | 29.364 | 12 [1; 3] | 5.1 | Tropomyosin [16-252] |
| D3TKW0 | Alternative splicing factor ASF/SF2 | 26.564 | 4 [2; 2] | 13.6 | RNA recognition motif [9-75; 119-196] |
| D3TLF0 | Malate dehydrogenase | 35.988 | 8 [5; 4] | 11.4 | Ldh-1 N [5-153], Ldh-1 C [156-330] |
| D3TR86 | Aconitase | 85.538 | 19 [17; 2] | 31.5 | Aconitase domains [69-509; 588-718] |
| D3TM62 | Aspartate amino-transferase | 47.899 | 6 [5; 2] | 5 | Aminotran 1-2 [56-424] |
| D3TPQ0 | Vesicle coat complex COPI epsilon subunit | 34.783 | 3 [2; 3] | 31.3 | Coatomer E [10-296] |
| D3TMY6 | Ubiquitin/40S ribosomal protein S27a fusion protein | 25.64 | 4 [4; 3] | 27.4 | Ubiquitin [6-74; 82-150; 158-226] |
| D3TN40 | Proteasome subunit beta type | 29.548 | 5 [2; 3] | 16.8 | Proteasome [36-217]/Pr beta C [232-267] domains |
| D3TLG3 | Isocitrate dehydrogenase [NADP] | 46.222 | 9 [6; 4] | 18.5 | Iso-DH [5-400] |
| D3TMF0 | Flavin reductase | 22.338 | 4 [3; 2] | 22.3 | NADH[P]-binding domain [4-187] |
| D3TMZ1 | Endocytosis/signaling protein EHD1 | 60.89 | 9 [8; 5] | 17.4 | Dynamin N [58-218], EF-hand 4 [439-533] |
| D3TLM8 | Multifunctional chaperone | 28.082 | 10 [7; 6] | 12.6 | 14-3-3 domain [6-239] |
| Q0QHL4 | Pyruvate carboxylase | 40.949 | 10 [7; 5] | 28.7 | ATP binding [1-182]/C-terminal [197-304] domains |
| D3TQB5 | Pyruvate carboxylase | 83.415 | 15 [12; 4] | 5.4 | HMGL-[146-396]/Conserved carboxylase [436-636/lipoyl attachment [685-752] domains |
| D3TRM2 | Fumarase | 53.365 | 5 [6; 2] | 32.1 | Lyase [42-374], Fumarase C C-terminus [440-494] |
| D3TRK1 | Trehalose 6-phosphate phosphatase | 31.23 | 10 [6; 7] | 41.8 | Trehalose-phosphatase [34-257] |
| D3TPE1 | 60S ribosomal protein L13 | 24.499 | 7 [6; 6] | 12.1 | Ribosomal protein L13e [6-185] |
| D3TQS9 | Citrate synthase | 51.732 | 12 [11; 4] | 4.2 | Citrate synthase [73-451] |
| D3TMR1 | Chaperonin complex (TcP-1) beta subunit CCT2 | 57.92 | 9 [6; 9] | 42.1 | Cpn60-TCP-1 [34-525] |
| D3TRU0 | Glyceraldehyde-3-phosphate dehydrogenase | 35.531 | 7 [6; 2] | 24.7 | NAD binding [2-149]/C-terminal [154-311] domains |
| D3TP00 | Ribosomal protein S14b | 16.114 | 5 [5; 4] | 3 | Ribosomal protein S11 [29-147] |
| D3TQI6 | Uncharacterized conserved glycine-rich protein | 28.937 | 4 [3; 3] | 11.6 | N- [87-153]/C-terminal [170-243] domains |
| D3TLH4 | Glutamic pyruvate transaminase 2 | 61.804 | 11 [10; 4] | 41.5 | Class I and II domain [157-538] |
| D3TRY2 | Porin | 30.307 | 16 [16; 2] | 17.5 | Porin 3 [2-275] |
| D3TRK5 | Uncharacterized integral membrane protein | 31.464 | 7 [3; 7] | 18 | Uncharacterized integral membrane protein [30-276] |
| D3TLG2 | Methylmalonate-semi-aldehyde dehydrogenase | 56.072 | 9 [7; 2] | 8.3 | Aldedh [34-498] |
| D3TPN5 | Arginine kinase | 39.898 | 19 [16; 8] | 4.5 | N- [16-93]/C-terminal [114-356] domains |
| D3TQR2 | Glutamate dehydrogenase | 60.863 | 22 [20; 12] | 26.7 | Dimerisation domains [101-233; 252-466] |
| D3TS59 | Vacuolar H+-ATPase v1 sector subunit H | 54.401 | 10 [9; 3] | 3.6 | N-terminal [22-324]/C-terminal [329-447] domains |
| D3TMY2 | Alkyl hydro peroxide reductase | 27.215 | 7 [5; 2] | 20.2 | AhpC-TSA [56-189], 1-cysPrx C [209-242] |
| D3TMZ0 | Oligosaccharyl-transferase gamma subunit | 36.986 | 5 [4; 2] | 5.9 | OST3-OST6 domain [163-315] |
| D3TMF3 | Pyruvate kinase | 55.803 | 8 [4; 5] | 13.7 | Barrel [25-378]/alpha/beta [393-513] domains |
| D3TLF9 | NAD-dependent malate dehydrogenase | 35.308 | 13 [12; 6] | 17.1 | NAD binding [25-168]/C-terminal [170-334] domains |
| D3TRE7 | Eukaryotic release factor 1 | 49.084 | 3 [3; 3] | 3.4 | eRF1 domains-1 [7-141]/2 [145-277]/ 3 [280-418]/ |
| D3TRU3 | Catalase | 56.896 | 8 [3; 5] | 12.2 | Catalase domain [25-410] |
| D3TMG7 | Vacuolar H+-ATPase v1 sector subunit C | 44.183 | 14 [14; 2] | 3.4 | V-ATPase subunit C [4-373] |
| D3TLZ1 | ADP ribosylation factor 79F | 20.556 | 5 [3; 3] | 29.8 | Arf [4-177] |
| D3TN69 | Hsp70/Hsp90 organizing protein-like protein | 55.916 | 4 [3; 2] | 2.1 | TPR repeat [2-69; 175-241; 309-376] |
| D3TPG3 | Translation elongation factor EF-1 gamma | 49.32 | 10 [9; 2] | 2.4 | GST N- [2-78]/C-terminal [96-194] domains/EF1domain [271-376] |
| D3TR48 | 60s ribosomal protein L9 | 21.188 | 6 [4; 4] | 2.2 | Ribosomal protein L6 [12-85; 97-176] |
| D3TRD7 | Succinyl-CoA synthetase alpha subunit | 34.859 | 4 [4; 2] | 36.7 | CoA binding domain [38-131], CoA-ligase [184-309] |
| D3TLG9 | Succinyl coenzyme A synthetase flavoprotein | 72.151 | 14 [11; 5] | 2.1 | FAD binding [58-453]/C-terminal [508-659] domains |
| D3TP78 | Succinyl-CoA synthetase alpha subunit | 26.985 | 6 [3; 3] | 11.9 | CoA binding [38-131]/CoA-ligase [184-309] domains |
| D3TR12 | NADH-ubiquinone oxidoreductase NDUFS2/49 kDa subunit | 52.244 | 5 [4; 3] | 3.3 | Complex1 49kDa [191-461] |
| D3TNQ0 | ADP/ATP translocase | 32.788 | 23 [20; 14] | 13.9 | OGC domain [7-105, 112-208, 208-300] |
| D3TRQ0 | Heat shock protein cognate 5 | 75.82 | 17 [14; 5] | 12.8 | Hsp70 [58-657] |
| D3TSC9 | Filamin alpha | 90.441 | 32 [31; 3] | 13.2 | Filamin [324-421] |
| D3TN22 | Mit-Cytochrome c oxidase subunit Va COX6 | 16.559 | 4 [2; 4] | 14.1 | Cox5A [29-137] |
| D3TRV9 | Ribosomal protein S5a | 25.404 | 10 [9; 6] | 56.5 | Ribosomal S7 [72-227] |
| D3TRT1 | Putative Tm2-PB | 32.587 | 13 [11; 2] | 6.5 | Tropomyosin [48-284] |
| D3TLT8 | 3-hydroxyacyl-CoA dehydrogenase | 26.874 | 11 [7; 9] | 5.3 | adh short domain [5-181] |
| D3TQG7 | Alpha tubulin | 49.777 | 18 [15; 5] | 3 | Tubulin/FtsZ family GTPase [3-226]/C-terminal [263-393] domains |
| D3TR96 | ATP synthase subunit b | 26.819 | 15 [15; 8] | 49.9 | ATP synthase B [68-229] |
| Q2PQQ0 | Serine protease inhibitor 4 | 46.185 | 3 [3; 2] | 19.2 | Serpin [44-413] |
| D3TRY9 | Cytochrome c oxidase subunit IV COX5b | 20.836 | 4 [2; 2] | 20.4 | COX4 [40-181] |
| D3TR32 | 40S ribosomal protein SA | 30.121 | 9 [8; 5] | 7.4 | Ribosomal S2 [18-186] |
| D3TR09 | Mitochondrial succinate dehydrogenase [ubiquinone] iron-sulfur subunit | 33.566 | 10 [8; 2] | 13.9 | 2Fe-2S iron-sulfur cluster binding domain [48-155], 4Fe-4S dicluster domain [194-267] |
| D3TM22 | Vesicle coat complex COPII GTPase subunit SAR1 | 21.723 | 7 [6; 3] | 6.1 | Arf [7-192] |
| D3TLI1 | Troponin | 24.392 | 11 [11; 3] | 20 | Troponin [44-173] |
| D3TRP1 | Multifunctional chaperone | 29.072 | 12 [9; 5] | 5.3 | 14-3-3 domain [4-239] |
| D3TSA7 | Glutathione S-transferase | 23.573 | 5 [4; 3] | 4.1 | GST N- [1-74]/C-terminal domains [124-185] |
| D3TPS8 | Calmodulin | 18.053 | 6 [6; 2] | 19.7 | EF-hand domain pair [15-76; 91-149] |
| Q0QHK6 | 1-pyrroline-5-carboxylate dehydrogenase 2 | 58.186 | 18 [17; 6] | 30 | Aldedh [53-516] |
| D3TSA4 | Hypothetical conserved protein | 32.035 | 3 [2; 2] | 51 | Farnesoic acid 0-methyl transferase [34-134] |
| D3TSB1 | Mitochondrial isocitrate dehydrogenase [NAD] | 38.39 | 8 [6; 2] | 12.7 | Isocitrate/isopropylmalate dehydrogenase domain [26-350] |
| D3TP19 | Heat shock protein 70 | 70.186 | 15 [8; 10] | 48.4 | HSP70 [3-607] |
| D3TRW4 | ATP synthase subunit beta | 54.448 | 23 [22; 17] | 51.4 | ATP synthase nucleotide binding domain [164-384] |
| D3TPL1 | Heat shock protein cognate 4 | 60.71 | 21 [19; 10] | 18.6 | HSP70 [1-516] |
| D3TPI6 | 40S ribosomal protein S4 | 27.126 | 8 [4; 8] | 39.6 | RS4NT [NUC023] domain [1-22], S4 domain [24-72] |
| D3TQL8 | 60s acidic ribosomal protein P0 | 33.789 | 8 [6; 7] | 7.6 | Ribosomal L10 [5-106], Ribosomal 60s [231-310] |
| D3TLB6 | Annexin | 35.491 | 7 [4; 2] | 50.2 | Annexin [19-84; 91-156, 174-240, 250-315] |
| D3TNF0 | Electron transfer flavoprotein beta subunit | 27.118 | 12 [12; 6] | 6.5 | Electron transfer flavoprotein domain [24-188] |
| D3TMF9 | 26S proteasome regulatory complex ATPase RPT1 | 48.461 | 6 [3; 4] | 11.5 | ATPase AAA domain [212-345] |
| D3TMC2 | 60s ribosomal protein L10A | 24.406 | 5 [4; 3] | 8.5 | Ribosomal L1 [4-213] |
| D3TR30 | tubulin beta-1 chain | 50.03 | 20 [12; 16] | 19.7 | Tubulin domains [3-224; 261-383] |
| D3TQ38 | PolyA-binding protein | 71.148 | 10 [6; 4] | 24.6 | RBD domains [4-74; 92-161; 185-254; 289-358] |
| D3TRS8 | Cytochrome c oxidase subunit VIb COX12 | 10.788 | 3 [2; 2] | 4.1 | Vib domain [28-89] |
| D3TPW0 | Histone H2A | 14.879 | 6 [3; 6] | 5.2 | Core histone H2A domain [20-94] |
| D3TQN0 | 40S ribosomal protein S3a | 30.403 | 13 [8; 10] | 43 | Ribosomal S3Ae domain [12-225] |
| D3TRH2 | Heat shock protein cognate 3 | 72.512 | 25 [22; 4] | 17.2 | HSP70 [31-636] |
| D3TPY1 | Oligosaccharyl-transferase delta subunit | 71.628 | 7 [7; 3] | 30.7 | Ribophorin II [4-633] |
| D3TMP5 | Putative dehydrogenase | 42.762 | 4 [2; 2] | 13.2 | short chain dehydrogenase domain [96-267] |
| D3TR05 | Heat shock protein 60 | 60.659 | 19 [17; 6] | 5.4 | cpn60-TCP-1 domain [43-546] |
| D3TRE3 | Disulfide-isomerase | 57.309 | 34 [33; 8] | 26.5 | Thioredoxin domain [29-133; 162-346; 369-473] |
| D3TLD9 | Putative aminopeptidase | 54.838 | 12 [11; 2] | 6.4 | Peptidase M17 [170-494] |
| D3TRW2 | Metallo-exopeptidase | 53.553 | 7 [7; 4] | 46.8 | Peptidase M20 [211-373] |
| D3TLL7 | Vesicle-associated membrane protein-associated protein A | 27.986 | 7 [7 ;5] | 5.6 | Major sperm protein domain [12-117] |
| D3TN04 | Alkyl hydro peroxide reductase | 26.477 | 8 [8; 2] | 4.9 | AhpC/TSA [44-177]/C-terminal [197-234] domains |
| D3TKZ1 | Dolichy diphospho-oligosaccharide glycosyl-transferase subunit 1 | 51.98 | 5 [5; 3] | 27.8 | Ribophorin I [23-437] |
| D3TLF8 | Putative histone tail methylase | 48.383 | 14 [13; 5] | 23.2 | SET domain [34-254] |
| D3TMR0 | Mitochondrial F1F0-ATP synthase (OSCP/ATP5) | 22.031 | 14 [12; 4] | 11.2 | ATP synthase delta [OSCP] domain [28-200] |
| D3TS01 | Nucleoside diphosphate kinase | 18.715 | 4 [3; 2] | 4.2 | Nucleoside diphosphate kinase domain [21-155] |
| D3TN53 | Clathrin adaptor complex medium subunit | 57.825 | 6 [6; 2] | 12.1 | Clathrin adaptor complex small chain domain [2-141] |
| D3TQ28 | Myosin class II heavy chain | 72.781 | 60 [52; 42] | 25.9 | Myosin tail 1 [1-600] |
| D3TSB0 | Protein disulfide-isomerase | 55.5 | 16 [15; 2] | 60.2 | Thioredoxin [20-126; 155-340; 362-467] |
| D3TMY7 | 40S ribosomal protein S20 | 13.365 | 4 [4; 2] | 23.5 | Ribosomal S10 [22-118] |
| D3TM80 | Elongation factor Tu | 53.711 | 10 [7; 2] | 53.8 | GTP binding [79-272]/2 [295-364]/C-terminal [368-468] domains |
| D3TRU4 | Ribosomal protein S3 | 29.503 | 10; 8; 6] | 3.1 | KH [42-118]/C-terminal [126-210] domains |
| D3TM57 | Glycogenin | 52.975 | 12 [12; 3] | 47.5 |  |
| D3TQ00 | Myosin heavy chain | 87.186 | 100 [89; 64] | 35.8 | Myosin tail 1 domain [1-724] |
| D3TLX2 | Thioredoxin/protein disulfide isomerase | 47.421 | 9 [8; 4] | 10.4 | Thioredoxin [26-130; 153-260] |
| D3TLM6 | NADH-ubiquinone oxidoreductase NDUFS3/30kDa subunit | 29.93 | 9 [9; 5] | 2.1 | Respiratory chain NADH dehydrogenase complex 1 30kDa subunit domain [94-199] |

TcP-1, Chaperonin complex component; W2 domain, Domain at the C-termini of guanine nucleotide exchange factor (GCD)-6, and elongation initiation factor (eIF) -2B epsilon/-4 gamma/-5; RS4NT, N-terminal domain of Ribosomal proteins S4/S3e proteins; SET domain, SET (Su(var)3-9, Enhancer-of-zeste, Trithorax) domain; KH, K homology RNA-binding domain; FtsZ, filamenting temperature-sensitive mutant Z family domain; MTC, mitochondrial tricarboxylate carrier.

**Table S 3: Annotation of eighteen host proteins that were down-regulated in GpSGHV-infected SG of *G. pallidipes* but up-regulated in the proteome of *G. m. morsitans*:** The number of unique peptides that identified the proteins in *G. m. morsitans* and *G. pallidipes*, respectively, are shown in the square brackets in column 4. The amino acid coordinates of the signature domains in the protein sequences are indicated in the square brackets in column 6. The 18 proteins listed in this table are also depicted in Figure 4, Panel C.

| **UniProt ID** | **Protein Name/Description** | **Mol. Weight [kDa]** | **Peptides [Unique]** | **Sequence Coverage [%]** | **Predicted signature/conserved domains; [amino acid coordinates in the protein sequence]** |
| --- | --- | --- | --- | --- | --- |
| D3TRX7 | Hypothetical conserved protein | 18.487 | 7 [6; 4] | 5.3 |  |
| D3TN39 | 26S proteasome regulatory complex ATPase RPT3 | 46.377 | 4 [2; 2] | 7.3 | ATPase [193-326] |
| D3TSC7 | Vacuolar H+-ATPase v1 sector subunit B | 54.083 | 25 [21; 13] | 47.7 | ATPase, F1 complex alpha/beta subunit [27-93], N-terminal domain P-loop containing nucleoside triphosphate hydrolase [95-384], ATPase, F1/V1/A1 complex, alpha/beta subunit, C-terminal [395-479] |
| D3TLI6 | Vacuolar H+-ATPase v1 sector subunit A | 68.093 | 26 [24; 8] | 10.4 | ATPase, F1 complex alpha/beta subunit, N-terminal domain [13-84], ATPase, F1/V1/A1 complex, alpha/beta subunit, nucleotide-binding domain [226-452], ATPase, F1/V1/A1 complex, alpha/beta subunit, C-terminal [467-613] |
| D3TLR6 | Vacuolar H+-ATPase v1 sector subunit D 1 | 27.602 | 7 [7; 2] | 8.4 | ATP synthase subunit D [14-208] |
| D3TR91 | Mitochondrial phosphate carrier protein | 38.33 | 9 [5; 2] | 60.2 | Mitochondrial carrier domain [55-329] |
| D3TP14 | Electron transfer flavoprotein alpha subunit | 34.497 | 17 [16; 4] | 4 | ETF [21-175], ETF alpha [210-295] |
| D3TLB1 | Vacuolar H+-ATPase v1 sector subunit E | 26.002 | 11 [11; 5] | 7.5 | vATP synthase [18-216] |
| D3TS74 | 60S ribosomal protein L8 | 27.847 | 7 [5; 4] | 17.2 | RNA binding [1-97]/2 [90-169]/3 [170-246] domains |
| D3TR42 | ATP synthase subunit alpha | 59.358 | 32 [21; 21] | 6.6 | ATPase [47-134]/P-loop containing nucleoside triphosphate hydrolase [136-421]/F1/V1/A1 C-terminal [422-550] domains |
| D3TR98 | Cytochrome b-c1 complex subunit Rieske, mitochondrial, isoform X1 | 27.577 | 6 [4; 3] | 5 | TM [64-129]/Rieske [160-250] domains |
| D3TR24 | 40S ribosomal protein S7 | 22.055 | 10 [8; 5] | 8.2 | Ribosomal S7e [4-191] |
| D3TM34 | Succinyl-CoA ligase subunit beta | 48.905 | 11 [11; 5] | 59.4 | ATP grasp 2 [40-248]/CoA ligase [307-427] domains |
| D3TMX6 | ATP synthase subunit gamma | 32.912 | 11 [9; 8] | 8.4 | ATP synthase [28-297] |
| D3TRB1 | Mitochondrial processing peptidase beta subunit | 50.391 | 19 [2; 12] | 8.5 | Peptidase M16 domains [33-180; 185-369] |
| D3TLB8 | Prohibitin | 30.193 | 12 [12; 9] | 6.4 | SPFH domain [28-209] |
| D3TRR8 | 60S ribosomal protein L3 | 46.609 | 6 [3; 3] | 20.7 | Ribosomal L3 [50-348] |
| D3TP61 | Actin filament-coating protein tropomyosin-2, isoform X12 | 32.641 | 25 [13; 6] | 32.7 | Tropomyosin [65-283] |

SPFH domain, named after proteins stomatin/prohibitin/flotillin/high frequency of lysogenization (Hfl)-K/C

**Table S 4: Annotation of nine host proteins that were down-regulated in both GpSGHV-infected SG proteomes of both *G. m. morsitans* and *G. pallidipes*:** The number of unique peptides that identified the proteins in *G. m. morsitans* and *G. pallidipes*, respectively, are shown in the square brackets in column 4. The amino acid coordinates of the signature domains in the protein sequences are indicated in the square brackets in column 6. The 9 proteins listed in this table are also depicted in Figure 4, Panel D.

| **UniProt ID** | **Protein Name/Description** | **Mol. Weight [kDa]** | **Peptides [Unique]** | **Sequence Coverage [%]** | **Predicted signature/conserved domains; [amino acid coordinates in the protein sequence]** |
| --- | --- | --- | --- | --- | --- |
| D3TQK0 | Actin 5C | 41.69 | 22 [3; 2] | 10.2 | Actin [6-376] |
| D3TS67 | Hypothetical protein | 39.269 | 9 [6; 5] | 25.5 |  |
| D3TNM9 | Signal peptidase complex subunit | 20.166 | 6 [6; 2] | 18.6 | SPC22 [1-175] |
| D3TPT6 | Actin 87E | 41.7 | 26 [1; 1] | 4.7 | Actin [3-376] |
| D3TPJ2 | Ubiquinol cytochrome c reductase subunit QCR2 | 46.157 | 11 [9 ;3] | 10.8 | Peptidase M16 domains [43-187; 192-367] |
| D3TRW9 | Fau hypothetical conserved protein | 14.342 | 6 [5; 4] | 36.5 |  |
| D3TSC6 | Zeelin1 | 38.007 | 25 [7; 11] | 9.9 |  |
| D3TM02 | myosin light chain 2 | 23.772 | 6 [6; 4] | 24.2 | EF-hand domain pair [68-137, 138-213] |
| D3TNV6 | Medium-chain acyl-CoA dehydrogenase | 46.046 | 15 [14; 4] | 24.4 | Acyl-CoA dehydrogenase [34-147, 153-251, 261-412] |

**Table S 5: Annotations of one hundred eighty-nine proteins that were detectable in the proteome of GpSGHV-infected *G. m. morsitans* but not in the proteome of *G. pallidipes*:** Of these, 65.1% (n=123) were up-regulated, while the remaining 66 proteins were down-regulated. The abundance distribution of these proteins is indicated in Figure 2 and in the X-axis of Figure 4.

| **UniProt ID** | **Protein name [Description]** | **Mol. weight [kDa]** | **Peptides** | **Sequence coverage [%]** | **Predicted signature/conserved domains; [amino acid coordinates in the protein sequence]** |
| --- | --- | --- | --- | --- | --- |
| **Up-regulated proteins (*n* = 123)** | | | | | |
| D3TKP9 | Cytochrome P450 | 28.401 | 2 | 7 | Cytochrome P450 domain [22-244] |
| D3TQA5 | Proteasome subunit alpha type | 29.045 | 2 | 5 | Proteasome subunit A domains [5-217] |
| D3TP23 | Translocon-associated complex TRAP gamma subunit | 21.774 | 2 | 10 | TRAP-gamma domain [18-187] |
| D3TQG4 | Troponin | 16.789 | 2 | 13.5 | EF hand domain [11-72; 84-148] |
| D3TLY8 | Phosphomanno mutase | 29.187 | 3 | 11.9 | Phosphomannomutase [30-252] |
| Q2PZ05 | Lipophorin | 83.154 | 5 | 7.3 | Vitellogenin lipid transport domain [1-52]/DUF1943 [83-382]/DUF1081 [404-537] |
| D3TPR7 | Proline synthetase co-transcribed protein | 28.257 | 3 | 8.8 | Alanine racemase domain [9-246] |
| D3TN31 | Translation initiation factor 2 alpha subunit | 38.807 | 2 | 7.3 | S1 [12-87]/alpha subunit [128-242] domains |
| Q0QHK5 | Pyrroline-5-carboxylate reductase | 25.831 | 5 | 22 | F420-oxidoreductase [6-101]/Pyrroline-5-carboxylate reductase [162-247] domains |
| D3TN30 | Cytochrome c | 11.637 | 3 | 37.4 | Cytochromes c domain [8-107] |
| D3TMJ0 | Mitochondrial 28S ribosomal protein S30 | 65.722 | 2 | 11.7 | PDCD9 domain [4-442] |
| D3TM36 | Putative dehydrogenase | 27.31 | 3 | 12.2 | ADH domain [6-173] |
| D3TLG8 | Putative esterase | 32.522 | 2 | 7.5 | Alpha/beta hydrolase domain [27-278] |
| D3TQP5 | Glutathione S transferase | 25.123 | 4 | 17.2 | GST N- [6-84]/C-terminal [66-196] domains |
| D3TP89 | NADH-ubiquinone oxidoreductase NDUFA8/PGIV/19 kDa subunit | 19.995 | 6 | 38.5 | - |
| D3TP84 | Vacuolar H+ATPase v1 sector subunit G | 13.769 | 5 | 35.3 | - |
| D3TLH5 | Translocation protein 1 | 45.109 | 2 | 5.3 | Sec62 domain [90-327] |
| D3TQS8 | Integrin beta | 27.34 | 3 | 12.5 | Integrin beta domain [42-247] |
| D3TKT6 | Synaptosomal-associated protein | 22.623 | 2 | 23.6 | SNAP 25 [100-151]/SNARE [155-203] domains |
| D3TNS8 | NADH-ubiquinone oxidoreductase NUFS7/PSST/20 kDa subunit | 24.297 | 2 | 8.2 | Oxidoreductase domain [94-204] |
| Q8IS37 | Nitric oxide synthase | 17.168 | 3 | 19.6 | Oxidoreductase NAD-binding domain [1-106] |
| D3TLE5 | FKBP-type peptidylprolyl *cis*-trans isomerase | 46.218 | 2 | 5.9 | FKBP [93-186]/Tetratricopeptide repeat [301-334] domains |
| L7P6U7 | Aquaporin | 30.881 | 2 | 10.1 | Aquaporin-like domain [16-263] |
| A3FMN4 | Tsal2 form B | 43.836 | 26 | 63.6 | DNA/RNA non-specific endonuclease domain [139-369] |
| Q694A8 | Putative peroxiredoxin | 17.961 | 4 | 31.5 | Redoxin domain [14-164] |
| D3TQ57 | Seleno-protein T | 22.246 | 2 | 14.8 | Rdx [41-182] |
| D3TM07 | Chloride intracellular channel | 29.447 | 4 | 18.8 | Thioredoxin [17-107]/GST C-terminal [115-230] domains |
| D3TL85 | Ribosomal protein S25 | 13.083 | 3 | 33.6 | S25 ribosomal protein domain [1-111] |
| D3TMC1 | Eukaryotic translation initiation factor 3 subunit F | 31.073 | 3 | 12.9 | JAB [4-112]/MitMem-regulation [158-275] domains |
| D3TPJ4 | Thioredoxin binding protein tBP2/VDUP1 | 45.814 | 2 | 6.2 | Arrestin N- [6-155]/C-terminal [178-310] domains |
| D3TMJ7 | Ras-related small GTPase rho type | 26.916 | 4 | 16.8 | Ras domain [7-154; 147-227] |
| D3TMK4 | Signal recognition particle receptor alpha subunit | 68.503 | 3 | 7.1 | N- [26-275]/helical bundle [297-370]/GTPase [399-618] domains |
| D3TRU6 | Carbonnitrogen hydrolase | 62.804 | 9 | 15.2 | Carbon-nitrogen hydrolase domain [49-229] |
| D3TR64 | G protein alpha subunit | 40.353 | 2 | 6.2 | G protein alpha subunit domain [3-343] |
| D3TNI0 | Hypothetical conserved protein | 47.749 | 2 | 5.1 | Ecdysteroid kinase domain [42-326] |
| D3TSD2 | Cytoplasmic tryptophanyl tRNA synthetase | 48.235 | 3 | 8.4 | tRNA synthetases class I domain [109-401] |
| D3TRB5 | Mitochondrial processing peptidase beta subunit | 52.749 | 16 | 41.4 | M16 [53-200] and inactive [205-387] domains |
| D3TME3 | Glucose-6-phosphate isomerase | 63.669 | 4 | 8.4 | Phosphoglucose isomerase domain [59-551] |
| D3TP83 | Diacetyl reductase/Lxylulose reductase | 25.523 | 3 | 11.3 | Short-chain dehydrogenase domain [8-163] |
| D3TP68 | Ca^2+^-binding protein | 23.052 | 2 | 9.4 | EF hand domain [34-101; 106-167] |
| D3TNY9 | Putative translation initiation inhibitor UK114/IBM1 | 14.556 | 2 | 18.2 | Endoribonuclease L-PSP domain [11-129] |
| D3TLF7 | Cysteinyl tRNA synthetase | 61.551 | 4 | 9.9 | tRNA synthetases class I [C]/catalytic [51-352] domains |
| D3TM50 | Kynurenine 3 monooxygenase | 52.016 | 3 | 6.6 | Monooxygenase, FAD-binding [19-372] |
| D3TKS6 | Translocase of outer membrane 70 | 60.325 | 3 | 6.9 | Tetratricopeptide helical domains [82-154; 248-395; 442-509] |
| D3TQT6 | Glycerol 3 phosphate dehydrogenase | 38.973 | 2 | 5.3 | NAD[P]-binding [2-193]/C-terminal [194-351] domains |
| D3TMH7 | Dihydropteridine reductase dHPR/QdPR | 24.973 | 3 | 14.1 | Short-chain dehydrogenase domain [4-160] |
| D3TMK2 | Ras-related small GTPase rho type | 21.158 | 2 | 12.1 | Ras domain [5-178] |
| Q9U7C6 | Salivary gland growth factor-1 | 56.5 | 40 | 57.6 | Adenosine deaminase N-terminal domain [7-100; 103-476] |
| D3TPD2 | Heme oxygenase | 31.801 | 3 | 11 | Heme-oxygnesase domain[15-242] |
| D3TKL8 | Stromal cell derived factor 2 | 24.319 | 2 | 9.7 | MIR domain [47-197] |
| D3TLR9 | Triosephosphate isomerase | 26.94 | 4 | 24 | Triosephosphate isomerase domain [5-243] |
| D3TS34 | Mitochondrial carnitineacylcarnitine carrier protein | 32.508 | 2 | 6 | Mitochondrial carrier domain [7-105; 107-202; 206-299] |
| D3TPU2 | Ubiquitin regulatory protein UBXD2 | 59.375 | 4 | 8.7 | Ubx protein domain [308-391] |
| D3TRN3 | Hypothetical conserved protein | 72.215 | 4 | 9.8 | Protein of unknown function [DUF3736] [240-372] |
| D3TMY3 | Pyruvate dehydrogenase E1 alpha subunit | 44.405 | 8 | 17.9 | Dehydrogenase E1 domain [74-369] |
| D3TKN1 | Ubiquitin Chydrolase | 22.819 | 2 | 10.4 | Peptodase C12 domain [1-185] |
| D3TPS2 | Calmodulin | 16.679 | 2 | 16.9 | EF hand domain [7-76; 85-146] |
| Q2PZ06 | Lipophorin | 94.557 | 11 | 18.8 | von Willebrand factor type D domain [269-412] |
| D3TLG7 | Adaptor protein complex AP2 mu1 | 49.869 | 3 | 6.7 | Clathrin adaptor [1-137]/medium subunit [157-437] domains |
| D3TLY5 | Salivary secreted protein | 29.568 | 4 | 23.4 | Immunoglobulin I-set domain [35-121; 118-206] |
| D3TM69 | Calreticulin | 46.808 | 7 | 25.4 | Calreticulin domain [29-340] |
| D3TM83 | Signal recognition particle subunit SRP72 | 73.973 | 5 | 11.2 | SRP72 RNA-binding domain [528-589] |
| D3TLY4 | NADH-ubiquinone oxidoreductase NDUFA10/42kDa subunit | 46.177 | 6 | 17.4 | Deoxynucleoside kinase domain [148-314] |
| D3TMG5 | Mitochondrial oxoglutarate/malate carrier protein | 31.227 | 2 | 6 | Mitochondrial carrier domain [12-96; 93-191; 194-283] |
| D3TR61 | Myosin alkali light chain 1 | 17.589 | 3 | 17.5 | EF hand domain [26-147] |
| D3TMM3 | Vacuolar H+ATPase v0 sector subunit D | 39.674 | 7 | 28.1 | ATP synthase [C/AC39] subunit domain [14-348] |
| D3TMT7 | Peptidylprolyl cistrans isomerase | 23.585 | 3 | 15.5 | Cyclophilin-like domain [57-220] |
| D3TLJ8 | Dihydrolipoamide Sacetyltransferase | 55.337 | 7 | 13.9 | Biotin attachment [73-147]/e3 binding [223-260]/catalytic [278-510] domains |
| L0LBH8 | Cytochrome c oxidase subunit 2 | 26.172 | 6 | 31 | TM domain [1-91]/Cupredoxin [92-225] domains |
| D3TLM5 | Adenylate kinase | 27.086 | 8 | 38.2 | P-loop containing nucleoside triphosphate hydrolase [27-241] |
| D3TRU2 | Putative bifunctional leukotriene A4 hydrolase/aminopeptidase LTA4H | 69.253 | 4 | 9.2 | Aminopeptidase N- [16-385]/C-terminal [464-612] domains |
| D3TNG0 | Ca2+/calmodulindependent protein phosphatase | 21.829 | 5 | 36.2 | EF hand domain [16-133; 108-174] |
| D3TMK7 | Mitochondrial sulfide quinone oxidoreductase | 51.08 | 4 | 12.6 | FAD/NAD[P]-binding domain [49-346] |
| D3TMX5 | Reticulonlike protein | 26.179 | 3 | 15.4 | - |
| D3TMQ1 | EnoylCoA hydratase | 31.748 | 9 | 38.4 | ClpP/crotonase-like domain [29-292] |
| Q0QHL1 | Isocitrate dehydrogenase (NAD+) 1 | 43.145 | 7 | 21 | Isopropylmalate dehydrogenase-like domain [49-387] |
| D3TRT6 | Shortchain acylCoA dehydrogenase | 45.952 | 8 | 23.4 | Acyl-CoA dehydrogenase/oxidase, N-/ middle domain [38-275] and C-terminal [262-414] domains |
| D3TRU9 | Dihydrolipoyl dehydrogenase | 53.144 | 14 | 31.2 | FAD/NAD[P]-binding [40-356]/dimerisation [373-497] domains |
| D3TRE4 | Putative fumarylacetoacetate hydralase | 25.933 | 3 | 14.1 | Fumarylacetoacetase, C-terminal-related domain [18-232] |
| D3TNC5 | Retrotransposon protein | 27.968 | 5 | 26.4 | Gag [83-171]/CCHC-type [221-237] domains |
| D3TLL4 | SuccinylCoA:3ketoacidcoenzyme A transferase | 55.238 | 15 | 35.3 | Subunit A [39-267]/subunit B [300-506] domains |
| D3TMY5 | Mitochondrial F1F0ATP synthase subunit delta/ATP16 | 16.638 | 4 | 30.3 | delta/epsilon; N-[17-113]/ C-terminal [113-154] domains |
| D3TN18 | Mitochondrial F1F0ATP synthase subunit D/ATP7 | 20.491 | 8 | 41.3 | - |
| D3TS17 | Mitochondrial phosphate carrier protein | 38.808 | 7 | 16.4 | Mitochondrial carrier domain [59-332] |
| D3TRM9 | Superoxide dismutase CuZn | 15.639 | 7 | 52.6 | Copper/zinc binding domain [3-151] |
| D3TN14 | Membrane traffic protein | 21.433 | 3 | 13.9 | - |
| D3TP02 | NADHubiquinone oxidoreductase NDUFB5/SGDH subunit | 21.918 | 4 | 19.6 | - |
| D3TSE2 | FKBPtype peptidylprolyl cistrans isomerase | 23.24 | 4 | 20.6 | FKBP [33-127]/EF-hand pair [135-202] domains |
| D3TRK8 | Adenylosuccinate synthase | 51.208 | 8 | 21.1 | P-loop containing nucleoside triphosphate hydrolase [26-461] |
| D3TS81 | Mitochondrial F1F0ATP synthase subunit E | 8.7802 | 3 | 21.8 | - |
| D3TKT5 | Salivary nitric oxide synthase | 38.57 | 7 | 21.6 | N-terminal domain [221-347] |
| D3TLV9 | Phosphoglycerate kinase | 43.861 | 5 | 14.3 | N- [4-188]/C-terminal [194-400] domains |
| D3TN23 | Lon protease homolog mitochondrial | 118.81 | 2 | 1.9 | Peptidase S16, lon N-terminal [100-467]/AAA+ ATPase domain [572-857]/RP S5 domain 2-type fold [836-1045] |
| D3TNX3 | Angiopoietinlike salivary protein | 39.727 | 8 | 29 | Fibrinogen, alpha/beta/gamma chain, C-terminal globular domain [131-348] |
| D3TP69 | 60s acidic ribosomal protein P1 | 11.162 | 2 | 35.8 | - |
| D3TRD6 | Glutathione Stransferase | 25.838 | 6 | 22.4 | Thioredoxin-like fold [3-91]/GST C-terminal [86-225] |
| D3TPA0 | Cytochrome C1 | 33.623 | 9 | 38.9 | c-like [68-261]/TM anchor C-terminal [261-306] domains |
| D3TMA2 | Putative GTpbinding protein | 44.804 | 2 | 6.6 | P-loop containing nucleoside triphosphate hydrolase [22-325] |
| D3TMQ7 | Dipeptidyl peptidase III | 82 | 3 | 4.2 | - |
| D3TSC5 | Calponin | 20.76 | 7 | 40.6 | Calponin homology domain [4-187] |
| D3TQW4 | Adenosine deaminaserelated growth factor C | 62.069 | 26 | 50.9 | Adenosine/AMP deaminase domains [29-117; 120-503] |
| D3TLZ8 | Rab protein geranylgeranyltransferase component A | 49.92 | 10 | 28.5 | - |
| D3TLI5 | Dopamine Nacetyltransferase | 27.091 | 4 | 24.8 | Acyl-CoA N-acyltransferase [20-217] |
| D3TLL3 | Phosphoglycerate mutase | 28.942 | 4 | 12.2 | - |
| D3TN25 | Golgi reassembly stacking protein GRASP65 | 52.286 | 5 | 14.5 | PDZ domain [12-204] |
| D3TMW6 | Aspartyl protease | 43.135 | 3 | 10.2 | Aspartic peptidase domain [28-394] |
| D3TNV2 | Enolase | 47.373 | 6 | 18.4 | Enolase N- [3-137]/C-terminal [128-432] domains |
| D3TMB8 | Superoxide dismutase Mn mitochondrial | 24.502 | 4 | 22.8 | Manganese/iron superoxide dismutase, N- [15-101] and C-terminal [103-213] domains |
| D3TLD3 | Vacuolar H+ATPase v0 sector accessory subunit S1 Ac45 | 43.236 | 2 | 5.7 | - |
| D3TRP2 | Putative transcriptional regulator DJ1 | 24.919 | 5 | 28 | Class I glutamine amidotransferase-like domain [43-224] |
| D3TNP4 | Elongation factor 1 beta/delta chain | 24.325 | 5 | 29.9 | EF1B domains [129-222] |
| D3TR70 | Dihydrolipoamide succinyltransferase | 51.288 | 5 | 14.1 | Single hybrid motif [81-174]/Chloramphenicol acetyltransferase-like [244-480] domains |
| D3TR71 | AcetylCoA acetyltransferase | 41.664 | 5 | 20.4 | Thiolase domains [6-267; 275-396] |
| D3TNM4 | Microtubulebinding protein | 19.645 | 5 | 40.4 | Mss4-like [1-172] |
| Q8WS65 | Ferritin 2 light chainlike protein | 24.904 | 12 | 60.2 | Ferritin/DPS protein domain [55-201] |
| D3TQR4 | Tricarboxylate transport protein | 34.577 | 6 | 18 | Mitochondrial carrier domain [35-312] |
| D3TLD0 | 3hydroxyisobutyrylcoenzyme A hydrolase | 41.916 | 4 | 11.5 | ClpP/crotonase-like domains [31-223; 277-347] |
| D3TLS2 | Mitochondrial NADHubiquinone oxidoreductase 24 kDa subunit | 26.698 | 7 | 36.1 | Thioredoxin-like fold [48-220] |
| D3TLN4 | Dihydrolipoamide transacylase alphaketo acid dehydrogenase E2 subunit | 51.047 | 3 | 8 | Biotin/lipoyl attachment domain [44-117]/2-oxoacid dehydrogenase acyltransferase/catalytic domain [233-460] |
| D3TLP6 | Molecular chaperone | 39.882 | 2 | 7.6 | DnaJ domains [16-119; 128-151; 192-328] |
| Q2PYZ6 | Ferritin | 23.282 | 8 | 43.6 | Ferritin/DPS protein domain [44-189] |
| D3TSE8 | NADH ubiquinone oxidoreductase NDUFS8/23 kDa subunit | 24.22 | 4 | 16.4 | Iron-sulphur binding domain [107-175] |
| D3TMM6 | Carnitine Oacyltransferase CPT2/YAT1 | 76.737 | 2 | 3.7 | - |
| **Down-Regulated Proteins (*n* = 66)** | | | | | |
| D3TR27 | Gammainterferon inducible lysosomal thiol reductase | 26.49 | 2 | 9.2 | - |
| Q694A7 | Putative peroxiredoxin | 24.665 | 8 | 37 | Thioredoxin-like fold [1-217] |
| D3TLR5 | Mitochondrial complement component 1Q subcomponent-binding protein | 29.694 | 4 | 11.8 | - |
| D3TS89 | Putative cargo transport protein ERV29 | 30.358 | 3 | 14.1 | - |
| D3TS61 | Dipeptidyl aminopeptidase | 72.384 | 9 | 20.1 | Peptidase S9B, N- [8-374]/ catalytic [382-634] domains |
| D3TM85 | Translocon-associated complex TRAP beta subunit | 21.306 | 3 | 19 | - |
| D3TS46 | Eukaryotic initiation factor 4B | 50.702 | 4 | 11.6 | Nucleotide-binding alpha-beta plait domain [79-157] |
| D3TLZ0 | EnoylCoA hydratase | 31.685 | 12 | 39 | ClpP/crotonase-like domain [32-292] |
| D3TNF4 | Signal recognition particle subunit srp54 | 55.669 | 4 | 10.5 | helical bundle [1-94]/GTPase [101-296]/M- [298-435] domains |
| D3TMH0 | Translation initiation factor 5A | 17.608 | 4 | 30.8 | Domain 2 [14-89]/C-terminal domain [85-154] |
| D3TLA9 | Small heat shock protein | 18.959 | 5 | 29.3 | Alpha crystallin/Hsp20 domain [9-146] |
| D3TRX0 | Hydroxysteroid dehydrogenaselike 2 | 43.539 | 3 | 8.1 | NAD[P]-binding domain [3-246], SCP2 sterol-binding domain [297-404] |
| D3TRX9 | Bcell receptorassociated protein 31 | 26.681 | 6 | 31 | - |
| D3TR49 | Verylongchain acylCoA dehydrogenase | 68.093 | 5 | 9.3 | N-/middle [61-311]/C-terminal [296-444] domains |
| D3TLF5 | Hsp70interacting protein | 44.122 | 3 | 11 | Tetratricopeptide-like helical domain [126-225] |
| D3TN03 | Headelevated expression protein | 23.309 | 4 | 18.6 | Cysteine alpha-hairpin motif [153-192] |
| D3TPU5 | Fructosebiphosphate aldolase | 39.685 | 6 | 26.7 | Aldolase-type TIM barrel [7-346] |
| D3TMX9 | Uncharacterized conserved protein | 33.764 | 2 | 10.3 | Tetratricopeptide-like helical domain [60-239] |
| D3TS87 | Tsal1 protein | 45.482 | 32 | 76.9 | DNA/RNA non-specific endonuclease [139-388] |
| D3TQW6 | Salivary secreted adenosine | 41.09 | 7 | 20.7 | Adenosine/AMP deaminase domains [20-103; 187-315] |
| D3TNB8 | Fe2+/Zn2+ regulated transporter | 36.47 | 2 | 3.2 | - |
| D3TR78 | Lectin | 19.631 | 12 | 61.3 | C-type lectin fold [16-152] |
| D3TQE0 | NADHubiquinone oxidoreductase NDUFA9/39kDa subunit | 47.161 | 8 | 26 | NAD[P]-binding domain [45-295] |
| D3TLM0 | Uncharacterized conserved protein | 28.908 | 7 | 39.8 | - |
| D3TPU1 | HydroxyacylCoA dehydrogenase/enoylCoA hydratase | 31.814 | 5 | 19.7 | ClpP/crotonase-like domain [32-278] |
| A3RGB2 | Putative salivary 5nucleotidase/apyrase | 100.07 | 9 | 15.5 | Calcineurin-like phosphoesterase domain, apaH type [27-340] |
| Q9U7C5 | Salivary gland growth factor2 | 58.091 | 22 | 40.2 | Adenosine/AMP deaminase domains [20-97; 100-481] |
| D3TPR9 | Glutathione Stransferase | 25.34 | 3 | 14 | Thioredoxin-like fold [1-88]/GST, C-terminal-like [82-213] |
| D3TLJ1 | Bifunctional ATP sulfurylase adenosine 5phosphosulfate kinase | 72.305 | 3 | 6.1 | P-loop containing nucleoside triphosphate hydrolase [32-234], PUA-like domain [240-400], Sulphate adenylyltransferase catalytic domain [405-638] |
| D3TLP3 | Glutathione Stransferase | 27.983 | 10 | 43.8 | Thioredoxin-like fold [4-86], GST C- domain [86-230] |
| D3TQN2 | Mitochondrial transcription factor A CG4217PA | 30.063 | 3 | 9.8 | High mobility group box domains [46-136; 144-241] |
| D3TKP7 | Nitricoxide synthase | 59.314 | 8 | 22.6 | Flavoprotein-like domains [1-148; 162-434] |
| D3TMP8 | Heat shock protein 23 | 22.899 | 7 | 46.1 | Alpha crystallin/Hsp20 domain [72-176] |
| D3TNI6 | Superoxide dismutase CuZn | 22.811 | 2 | 10.2 | Copper/zinc binding domain [41-184] |
| D3TSB3 | Metallopeptidase | 43.714 | 10 | 28.4 | Peptidase M24, structural domain [3-336] |
| D3TN88 | Alpha crystallin | 20.41 | 6 | 45.9 | Alpha crystallin/Hsp20 domain [50-154] |
| D3TLK6 | Salivary gland growth factor1 | 56.459 | 42 | 59.4 | Adenosine/AMP deaminase domains [22-100; 104-474] |
| A3FMN3 | Tsal2 protein | 43.87 | 26 | 66.1 | DNA/RNA non-specific endonuclease [141-369] |
| D3TME4 | Cytochrome c oxidase subunit Vb COX4 | 13.571 | 2 | 20.2 | - |
| D3TLT7 | Hypothetical conserved protein | 62.851 | 5 | 11.5 | - |
| D3TRT5 | Rab protein 5 | 24.362 | 5 | 25.3 | P-loop containing nucleoside triphosphate hydrolase [25-221] |
| D3TLC3 | WW domaincontaining protein | 29.041 | 3 | 12.8 | PDZ domain [8-98] |
| D3TS33 | NADH dehydrogenase flavoprotein 1 ubiquinone | 51.427 | 8 | 20.3 | FMN-binding [95-265]/Soluble ligand binding [291-340]/Fe-S binding [379-424] domains |
| Q95P65 | 5'-nucleotidase family salivary protein | 61.932 | 16 | 26.9 | ApaH type [28-340]/C-terminal [344-554] domains |
| D3TMD3 | Rab protein 14 | 24.23 | 2 | 9.8 | P-loop containing nucleoside triphosphate hydrolase [4-198] |
| Q9NBA6 | Salivary antigen 5 variant | 28.793 | 14 | 43.8 | CAP domain [23-220] |
| D3TSM2 | Putative membrane protein | 36.044 | 8 | 23 | - |
| D3TR28 | Calponin | 20.021 | 11 | 58.5 | Calponin homology domain [10-170] |
| D3TMW5 | Tsal2 protein | 43.824 | 26 | 63.6 | DNA/RNA non-specific endonuclease [139-369] |
| D3TQG3 | Hypothetical conserved protein | 15.736 | 3 | 31.5 | - |
| D3TR11 | Translocase of outer mitochondrial membrane complex subunit TOM20 | 18.957 | 3 | 33.3 | Tom20 domain [55-141] |
| D3TSQ1 | Hypothetical secreted protein | 21.901 | 4 | 22.3 | - |
| D3TQA6 | Rab protein 8 | 23.637 | 2 | 11.2 | Small GTP-binding protein domain [7-162] |
| K7YYZ7 | AlanyltRNA synthetase | 47.484 | 2 | 6.2 | Core domain [1-431] |
| D3TP39 | Mitochondrial aspartate/glutamate carrier protein aralar/citrin | 75.957 | 4 | 5.1 | EF hand domain pair [14-128; 140-245] |
| D3TQ70 | Proteasome subunit alpha type | 27.896 | 3 | 13.9 | Nucleophile aminohydrolases N-terminal domain [6-231] |
| D3TRC7 | Putative uncharacterized protein | 27.273 | 4 | 22.8 |  |
| D3TN80 | Hypothetical conserved protein | 26.512 | 2 | 12.8 | - |
| D3TQP8 | Putative peroxiredoxin | 24.866 | 2 | 8.1 | Thioredoxin-like fold [6-219] |
| D3TP16 | Putative uncharacterized protein | 52.317 | 2 | 4.7 | - |
| D3TRF1 | Hypothetical conserved protein | 33.175 | 2 | 11.8 | Single-stranded nucleic acid binding R3H domain [17-138] |
| D3TL18 | Salivary alkaline phosphatase | 44.25 | 4 | 10.1 | Alkaline-phosphatase-like core domain [2-362] |
| D3TR21 | AcylCoA synthetase | 80.22 | 2 | 3.1 | AMP-dependent synthetase domains [115-580; 590-676] |
| D3TL02 | Putative uncharacterized protein | 43.926 | 2 | 4.7 | TRAF-[62-198]/BTB-POZ fold [213-329] domains |
| D3TQU9 | Translocon-associated complex TRAP alpha subunit | 32.955 | 2 | 12.8 | - |
| A3RGB0 | Glycine/glutamaterich protein sgp1 | 10.415 | 3 | 25.7 | - |

**Table S 6: Annotation of one hundred thirty-three proteins that were detectable in the proteome of GpSGHV-infected *G. pallidipes* but not in the proteome of *G. m. morsitans***: Of these, 96.9% (n=129) were up-regulated, while the remaining 4 proteins were down-regulated. The abundance distribution of these proteins is indicated in Figure 3 and in the Y-axis of Figure 4.

| **UniProt ID** | **Protein name [Description]** | **Mol. weight [kDa]** | **Peptides** | **Sequence coverage [%]** | | **Predicted signature/conserved domains; [amino acid coordinates in the protein sequence]** |
| --- | --- | --- | --- | --- | --- | --- |
| **Up-regulated proteins (*n* = 129)** | | | | | | |
| D3TNK6 | DNA helicase TIP49 | 53.54 | 4 | 9.6 | TIP49 [17-408] | |
| D3TN10 | 26S proteasome regulatory complex subunit RPN5/PSMD12 | 57.316 | 4 | 7.6 | PCI [342-453] | |
| D3TRT7 | FK506binding protein | 42.515 | 7 | 15.1 | FK506 binding domain [290-381] | |
| D3TKY1 | ATPcitrate lyase | 77.722 | 4 | 6.6 | CoA binding [102-208]/ligase domain [268-393]/synthase [487-694] domains | |
| D3TP03 | Actin 57B | 41.687 | 25 | 70.7 | Actin [3-376] | |
| D3TP12 | Putative splicing factor | 21.68 | 6 | 31.6 | RRM1 [19-89] | |
| D3TLR1 | Transmembrane emp24 domain containing 3 | 25.339 | 3 | 12.2 | Emp24/gp25L domain [33-210] | |
| D3TS85 | 60s ribosomal protein L11 | 21.48 | 3 | 15.4 | Ribosomal_L5 domains [20-73; 77-176] | |
| D3TN70 | GTPbinding protein | 35.439 | 4 | 19.8 | Gtr1/RagA [4-230] | |
| D3TM84 | GTPase Ran/TC4/gSP1 | 24.565 | 7 | 42.1 | Ras [11-169] | |
| D3TPG0 | RNAbinding protein p54nrb | 67.178 | 8 | 15.6 | RRM1 [219-283; 293-359]. NOPS [364-416] | |
| D3TP71 | Pseudouridine synthase | 68.869 | 2 | 3.9 | DKCLD [NUC011] domain [46-104]. TruB_N [108-224]. PUA domain [295-368] | |
| D3TN58 | 26S proteasome regulatory complex subunit RPN3/PSMD3 | 56.154 | 12 | 25.3 | PCI domains [317-421; 424-491] | |
| D3TLP9 | Tcomplex protein 1 subunit alpha | 59.061 | 9 | 18.4 | Cpn60 TCP1 domain [31-538] | |
| D3TNG7 | NADHcytochrome b5 reductase | 35.232 | 2 | 8.6 | FAD- [56-163]/NAD-binding [189-298] domains | |
| D3TNY3 | GMP synthase | 76.445 | 9 | 17.9 | GATase [20-202]/NAD- [213-304]/GMP-synthase [480-682] domains | |
| D3TN05 | Serine hydroxymethyltransferase | 63.504 | 5 | 11.7 | Serine hydroxymethyltransferase domains [120-518] | |
| D3TQ41 | Small nuclear ribonucleoproteinassociated protein | 20.963 | 3 | 21.6 | LSM domain [7-82] | |
| D3TM00 | Eukaryotic translation initiation factor 3 subunit D | 62.842 | 2 | 4.2 | EIF-3_zeta domain [8-523] | |
| D3TMP9 | Heat shock protein 23 | 23.005 | 3 | 15 | HSP20 [80-176] | |
| D3TLK7 | Na+/K+ ATPase beta subunit | 35.868 | 2 | 5.4 | Na-K-ATPase domain [2-310] | |
| D3TP08 | Factin capping protein beta subunit | 31.16 | 4 | 19.6 | F-actin capping protein domain [5-240] | |
| D3TMI6 | RNA polymerase I transcription factor subunit Spp27 | 26.715 | 5 | 22.7 | DEK C [5-58]/SWIB-MDM2 [163-238] domains | |
| D3TNF8 | Proteasome subunit beta type | 30.871 | 3 | 11.2 | Proteasome [69-251] | |
| D3TPI0 | Suppressor of profilin 2 | 42.14 | 3 | 9.4 | WD40 [45-83; 136-174; 321-361] | |
| D3TLT0 | Molecular chaperone | 45.054 | 7 | 22.3 | DnaJ [6-65]. [137-203] | |
| D3TSE5 | 60s ribosomal protein L13a | 23.476 | 4 | 13.7 | Ribosomal L13 domain [8-124] | |
| D3TMS1 | Ribosomal protein L18 | 21.589 | 5 | 27.8 | Ribosomal_L18e [2-123] | |
| D3TNS1 | AICAR transformylase | 63.406 | 8 | 25.1 | MGS-like [16-130], AICARFT/IMPCHase [145-360] domains | |
| D3TNU2 | Proteasome activator subunit | 28.16 | 4 | 17.8 | Pa28 alpha [1-64] and beta subunit [99-248] domains | |
| D3TNN2 | Serine/threonineprotein phosphatase | 34.447 | 7 | 26.9 | Calcineurin-like phosphoesterase domain [55-250] | |
| D3TN59 | Secretory carrier membrane protein | 38.625 | 2 | 7.7 | SCAMP domains [120-299] | |
| D3TQ30 | Ribosomal protein S15Aa | 14.626 | 3 | 25.6 | Ribosomal S8 domain [5-130] | |
| D3TLV3 | Methylcrotonoylcoenzyme A carboxylase 1 alpha | 77.711 | 2 | 3.4 | Carbamoyl phosphate synthetase domain [29-138]. ATP binding domain [144-353]. Biotin carboxylase domain [365-472]. Biotin attachment domain [628-695] | |
| D3TM82 | Cell membrane glycoprotein | 41.329 | 3 | 8.3 | Proteasome Rpn13 domain [24-107] | |
| D3TL05 | ATPdependent RNA helicase | 72.579 | 7 | 12.6 | DEAD box [142-313]/Helicase C [383-460] domains | |
| D3TML1 | Glycine Nmethyltransferase | 33.096 | 2 | 5.9 | Methyltransferase domain [61-182] | |
| D3TND0 | Ribosome bioproteinsis protein Nop58p/Nop5p | 58.38 | 2 | 5.1 | NOP5NT [1-66]/NOSIC [162-214]/snoRNA binding [254-402] domains | |
| D3TQP7 | DNA helicase | 50.1 | 4 | 10.8 | Reptin or TIP49b domain [14-416] | |
| D3TNX4 | Selenophosphate synthetase | 43.414 | 5 | 18.6 | AIR synthase related protein N- [89-186] and C-terminal [214-387] domains | |
| D3TQ73 | Proteasome formation inhibitor PI31 | 30.266 | 4 | 14.3 | PI31 proteasome regulator N-terminal domain [12-160] | |
| D3TS28 | Differentiationrelated protein 1 protein | 39.245 | 3 | 11 | Ndr domain [22-303] | |
| D3TSB6 | Ubiquitinconjugating enzyme E2 | 16.305 | 2 | 12 | Ubiquitin-conjugating domain [15-139] | |
| D3TLA6 | Casein kinase II alpha subunit | 39.852 | 7 | 29.6 | Protein kinase domain [37-322] | |
| D3TMI7 | Geranylgeranyltransferase type II beta subunit | 38.939 | 2 | 4.6 | Prenyltransferase domains [82-197; 224-267; 272-316] | |
| D3TNE2 | Proteasome subunit alpha type | 26.468 | 2 | 9.1 | Subunit A domains [8-30; 31-220] | |
| D3TP13 | GTPase Rab4 | 23.481 | 3 | 19.7 | Ras domain [10-171] | |
| D3TR44 | Lanthionine synthetase Clike protein 1 | 47.664 | 2 | 6.7 | Lanthionine synthetase C-like domain [50-417] | |
| D3TQV5 | GTPbinding ADPribosylation factorlike protein | 21.142 | 2 | 12.4 | ADP ribosylation factor domain [7-181] | |
| D3TM11 | Phosphoribosylamidoimidazolesuccinocarboxamide synthase | 46.892 | 5 | 12.5 | SAICAR [9-252]/AIR carboxylase [262-410] domains | |
| D3TLF2 | Adenosine monophosphate deaminase | 89.244 | 6 | 10 | Adenosine deaminase domain [317-724] | |
| D3TLT4 | 26S proteasome regulatory complex subunit RPN7/PSMD6 | 45.36 | 5 | 13.9 | RPN7 [66-239]/PCI [254-358] domains | |
| D3TPN7 | COP9 signalosome subunit cSN4 | 46.447 | 5 | 11.6 | PCI domain [267-366] | |
| D3TRI9 | ssDNA-binding replication protein A medium subunit RPA 30kD | 26.591 | 3 | 11.2 | RPA domain [140-235] | |
| D3TSN9 | DUTPase | 19.775 | 2 | 12.7 | DUTP diphosphatase domain [29-158] | |
| D3TRQ9 | Aldoketo reductase | 35.775 | 3 | 12.7 | Aldo-keto reductase domain [17-293] | |
| D3TN09 | Proteasome subunit alpha type | 31.325 | 4 | 17.3 | Proteasome subunit A domains [6-28; 29-221] | |
| D3TRW6 | Ribosomal protein L30 | 12.134 | 2 | 25.5 | Ribosomal protein L7Ae domain [12-106] | |
| D3TPW6 | Actinrelated protein 87C | 42.042 | 3 | 6.7 | Actin [2-372] | |
| D3TME6 | ATPdependent RNA helicase | 78.419 | 5 | 7.5 | KH [70-128]/DEAD box [300-471]/C-terminal [539-616] domains | |
| D3TMH6 | Phosphoinositide phosphatase | 68.11 | 4 | 6.9 | SacI homology domain [61-354] | |
| D3TNQ6 | Lectin VIP36 | 37.12 | 3 | 12.1 | L-type lectin domain [28-253] | |
| D3TPM6 | Putative sugar kinase | 33.11 | 4 | 13.1 | CARKD domains [40-293] | |
| D3TNI4 | Phosphoglucomutase/phosphomannomutase | 60.491 | 6 | 15.1 | Alpha/beta/alpha I [47-180]/II [185-295]/C-terminal [450-526] domains | |
| D3TRZ7 | Aspartate aminotransferase | 45.838 | 3 | 10.1 | Aminotransferase class I and II domain [29-397] | |
| D3TN11 | G protein beta subunit | 36.915 | 2 | 5.9 | WD40 domain [46-125; 134-340] | |
| Q2PQQ6 | Fibrillarin protein | 29.124 | 4 | 18.3 | Fibrillarin domain [39-266] | |
| D3TNB6 | Serine/threonine protein kinase/TGF-beta stimulated factor | 36.288 | 3 | 13.6 | Protein kinase domain [28-307] | |
| D3TP92 | AcylCoA reductase | 59.077 | 4 | 10.3 | NAD binding 4 domain [19-219] | |
| D3TQ93 | Dolichylphosphate betaglucosyltransferase | 37.06 | 4 | 12.8 | Glycosyl transferase family 2 domain [67-248] | |
| D3TP34 | Ribosephosphate pyrophosphokinase | 41.83 | 4 | 13.2 | Ribose phosphate pyrophosphokinase domains [49-183; 262-377] | |
| D3TPE0 | Ras-related small GTPase rho type | 21.325 | 3 | 13.6 | Ras domain [5-178] | |
| D3TMG2 | 60s ribosomal protein L6 | 25.947 | 3 | 14.7 | Ribosomal protein L6e domain [114-225] | |
| D3TRA6 | ATPdependent RNA helicase | 67.08 | 5 | 10.9 | DEAD/DEAH box helicase domain [157-328]. Helicase conserved C-terminal domain [400-477] | |
| D3TNZ7 | Nucleotide excision repair factor NEF2 RAD23 component | 41.032 | 3 | 8.5 | Ubiquitin [6-77]. UBA protein domain [173-209]. XPC-binding domain [257-315] | |
| D3TP51 | Putative dioxygenase | 33.123 | 4 | 15.7 | Memo-like protein domain [5-289] | |
| D3TN61 | RNAbinding protein Sam68 | 45.658 | 2 | 6.5 | K Homology [KH] domain [124-177] | |
| D3TKS0 | Trypsin | 28.427 | 3 | 10.8 | Tryp_SPc domain [29-251] | |
| D3TMR4 | Eukaryotic translation initiation factor 3 subunit L | 62.965 | 5 | 8.8 | RNA pol I-associated factor PAF67 domain [120-527] | |
| D3TLS6 | 26S proteasome regulatory complex subunit RPN6/PSMD11 | 47.087 | 4 | 8.6 | PCI domain [284-388] | |
| D3TS37 | Eukaryotic translation initiation factor 3 subunit H | 38.674 | 2 | 5.9 | JAB1/Mov34/MPN/PAD-1 ubiquitin protease domain [17-217] | |
| D3TPG9 | Replication factor C subunit RFC3 | 40.804 | 2 | 5.4 | DNA polymerase III delta subunit domain [18-188]. Replication factor C C-terminal domain [250-339] | |
| D3TRJ5 | Cytochrome bc1 complex subunit 7 | 13.42 | 3 | 25.5 | Ubiquinol-cytochrome C reductase domain [8-106] | |
| D3TS08 | Adenylosuccinate lyase | 54.534 | 6 | 14.7 | Fumarate lyase [21-309]. Adenylosuccinate lyase C-terminal domain [374-458] | |
| D3TQT5 | Pleiotropic regulator 1 | 53.287 | 3 | 5.5 | WD40 domain [158-454] | |
| D3TPQ1 | LysinetRNA ligase | 64.352 | 2 | 3.9 | OB-fold nucleic acid binding domain [98-179]. Aminoacyl tRNA synthetase domain [195-548] | |
| D3TRV5 | Eukaryotic translation initiation factor 3 subunit I | 36.045 | 2 | 8.6 | WD40 domain [4-37; 42-80; 179-217; 276-312] | |
| D3TL78 | Putative glycinrich protein | 21.048 | 2 | 12.7 | KH domain [147-209] | |
| D3TMJ5 | 26S proteasome regulatory complex ATPase RPT2 | 49.249 | 3 | 7.3 | AAA domain [221-354] | |
| D3TMJ4 | Guanine nucleotide binding protein alpha q polypeptide | 41.551 | 3 | 11.6 | G-alpha subunit domain [2-342] | |
| D3TLX5 | Eukaryotic translation initiation factor 3 subunit G | 30.124 | 2 | 10.5 | G-[22-140]/RNA recognition motif [189-258] domains | |
| D3TQX0 | Proliferating cell nuclear antigen | 28.942 | 2 | 6.2 | N- [1-125]/C-terminal [127-254] domains | |
| D3TLE9 | Mitogenactivated protein kinase kinase MKK3/MKK6 | 37.667 | 3 | 10.4 | Protein kinase domain [46-307] | |
| D3TMW2 | Proteasome subunit beta type | 30.469 | 2 | 7.7 | Proteasome domain [60-247] | |
| D3TMM1 | 26S proteasome regulatory complex subunit RPN11 | 34.337 | 2 | 8.8 | JAB [24-137] and MitMem regulation [170-293] domains | |
| D3TM53 | Putative dynamitin | 42.893 | 3 | 13.2 | - | |
| D3TPT3 | Putative cell surface protein-like protein | 49.325 | 2 | 6.1 | Surface antigen domain [130-443] | |
| D3TQD0 | 20S proteasome regulatory subunit beta type PSMB1/PRE7 | 25.798 | 2 | 14.9 | Proteasome subunit domain [26-221] | |
| D3TN42 | Alternative splicing factor SRP20/9G8 | 16.186 | 2 | 15.3 | RRM domain [13-78] | |
| D3TMG4 | 26S proteasome regulatory complex ATPase RPT5 | 47.52 | 2 | 4.9 | AAA domain [211-344] | |
| D3TM14 | Ubiquitin protein ligase | 17.063 | 3 | 22.7 | Ubiquitin-conjugating enzyme domain [7-144] | |
| D3TL34 | Ubiquinol cytochrome c reductase subunit QCR2 | 25.833 | 3 | 16.1 | Peptidase M16 inactive domain [8-183] | |
| D3TLH3 | Importin subunit alpha | 57.384 | 3 | 5.6 | Importin domain [2-91] | |
| D3TNM8 | NADPHcytochrome P450 reductase | 75.962 | 3 | 5.4 | Flavodoxin [80-217]/FAD-[272-491]/NAD-binding [525-636] domains | |
| D3TNU1 | Vacuolar sorting protein vPS45/Stt10 | 65.742 | 3 | 7.8 | Sec1 domain [22-550] | |
| D3TQ68 | Ubiquitin carboxylterminal hydrolase | 55.332 | 2 | 5.3 | Ubiquitin carboxyl-terminal hydrolase domain [103-474] | |
| D3TLW0 | RNAbinding protein musashi | 44.282 | 4 | 15.9 | RRM domain [9-78; 98-168] | |
| D3TQI0 | Exosomal 35 exo-ribonuclease complex subunit Rrp4 | 33.367 | 3 | 13.1 | RRP4 N-terminal domain [29-67] | |
| D3TMA4 | Actin-related protein ARP2/3 complex subunit ARPC2 | 34.872 | 4 | 15 | p34-Arc domain [56-286] | |
| D3TP04 | Cytochrome P450 | 57.675 | 2 | 4.2 | p450 domain [34-495] | |
| D3TQR9 | Serine/threonineprotein phosphatase | 37.61 | 4 | 14 | Metallophosphoesterase domain [56-251] | |
| D3TNA3 | Leucine carboxyl methyltransferase 1 protein | 37.962 | 2 | 7 | Leucine carboxyl methyltransferase domain [13-202] | |
| D3TL84 | Caspase | 37.198 | 2 | 6.9 | Peptodase C14 domain [88-322] | |
| D3TPL3 | Molecular chaperone prefoldin subunit 3 | 22.442 | 2 | 9.2 | Prefoldin domain [59-178] | |
| D3TPH9 | Hypothetical conserved protein | 42.914 | 2 | 4.4 | Ubiquitin [19-80] | |
| D3TS30 | Serine/threonineprotein phosphatase | 35.323 | 3 | 12.7 | Metallophosphoesterase domain [50-245] | |
| Q8MX87 | Transferrin | 71.9 | 3 | 5.9 | Transferrin domain [27-365; 373-466; 500-623] | |
| D3TR23 | Mitotic spindle checkpoint protein BUB3 | 37.411 | 2 | 5.5 | WD40 domain [3-42; 88-123; 127-163; 225-262] | |
| D3TQI4 | Ubiquitin-like protein activating enzyme N subunit | 38.358 | 2 | 8.5 | ThiF domain [44-176] | |
| D3TNB1 | Putative peroxiredoxin | 24.645 | 9 | 40.2 | AhpC/TSA domain [3-141] | |
| D3TQQ6 | Ca2+binding protein | 20.812 | 2 | 10.7 | EF hand domain [8-76; 79-142; 147-175] | |
| D3TR06 | Translation initiation factor 2 beta subunit | 35.897 | 2 | 7.3 | IF2B/IF5 domain [164-291] | |
| D3TNT4 | MAP kinase activity 2 | 41.773 | 2 | 5.6 | Protein kinase domain [20-307] | |
| D3TNG5 | Focal adhesion protein PINCH1 | 39.649 | 2 | 6.4 | LIM [18-75; 79-134; 143-206; 211-266; 270-326] | |
| D3TL17 | 26S proteasome regulatory complex subunit RPN10/PSMD4 | 38.859 | 2 | 4.2 | Ubiquitin interacting motif domain [177-194; 241-258; 268-285] | |
| D3TQW9 | 60s ribosomal protein L34 | 17.22 | 2 | 13.2 | Ribosomal protein L34e domain [1-94] | |
| D3TMI5 | Methylthioadenosine phosphorylase MTAP | 31.871 | 2 | 10.8 | Nucleoside phosphorylase domain [17-266] | |
| D3TPK9 | Uncharacterized conserved protein | 46.089 | 2 | 4.9 | FYVE zinc finger domain [286-360] | |
| D3TRQ2 | Ferritin | 24.918 | 12 | 60.2 | Ferritin domain [53-202] | |
| **Down-regulated proteins (n = 4)** | | | | | | |
| D3TS21 | 26S proteasome regulatory complex ATPase RPT6 | 45.51 | 6 | 21.3 | | AAA domain [184-317] |
| D3TPV3 | Fructosebisphosphate aldolase | 39.483 | 6 | 26.7 | | Aldolase-type TIM barrel [15-364] |
| D3TNK3 | Translocon-associated complex TRAP delta subunit | 18.298 | 2 | 11.9 | | TRAP-delta [4-169] |
| D3TQK7 | Antigen 5 | 28.824 | 8 | 27.5 | | CAP domain [59-207] |

**Table S 7: Annotation of fifty-eight GpSGHV proteins detected in the SG proteomes of *G. pallidipes* and *G. m. morsitans*:** The proteins listed are those that were identified with at least two peptides matching the same protein, of which at least one peptide was unique, and at least one peptide was unmodified. Where applicable, the homologies of the identified proteins to proteins in other viruses/microorganisms/cellular proteins are indicated in the square brackets in column 2. Gene Ontologies (GOs) are based on Blast2GO v 3.0.4 annotation (see article text for details). Only five GpSGHV proteins (shown in bold) were detectable in the SG proteome of *G. m. morsitans*.

| **UniProt ID** | **ORF Name/Protein Description [Homologies]** | **Mol. Weight [kDa]** | **Peptides [Unique]** | **Sequence Coverage [%]** | **Gene Ontology (GO) Annotation** | **Predicted conserved/signature domains (functional and/or structural)** |
| --- | --- | --- | --- | --- | --- | --- |
| B0YLF6 | SGHV001; [p74 protein-like protein; *Spodoptera pectinicornis* NPV] | 81.399 | 6 [5] | 10.2 | (BP):viral life cycle | TM; SP; isoleucine-rich activation motif |
| B0YLF7 | SGHV002 | 38.665 | 15 [14] | 41.9 |  |  |
| B0YLG1 | SGHV007 | 41.044 | 4 [2] | 11 |  |  |
| B0YLG2 | SGHV008 | 31.599 | 2 [2] | 11.1 |  |  |
| **B0YLG4** | **SGHV010; [ORF MSV156, *Melanoplus sanguinipes* EPV]** | **127.03** | **55 [36]** | **53.9** |  | **Coiled coils; Potential N-glycosylation sites; multiple serine/threonine/tyrosine-rich regions; Proline-rich profile; NLS-BP; Bromodomain-2 profile; PPASE-TENSIN** |
| **BOYLH2** | **SGHV018** | **8.642** | **2 [2]** | **14.7** |  | **ZFC3HC4 ring** |
| B0YLI4 | SGHV030 | 15.919 | 3 [2] | 26.3 | (BP):DNA integration; DNA recombination; (MF): DNA binding; | DNA breaking-rejoining enzyme (catalytic core) |
| B0YLI5 | SGHV031 | 33.604 | 5 [3] | 16.8 | (BP):DNA integration; DNA recombination; (MF): DNA binding | Leucine/isoleucine-rich regions; N-myristoylation sites; HDAC-interaction like domain protein |
| B0YLI7 | SGHV033 | 41.009 | 4 [4] | 11.2 |  |  |
| B0YLI8 | SGHV034; [ORFAMV260, *Amsacta moorei* EPV] | 41.179 | 3 [3] | 8.3 |  |  |
| B0YLJ0 | SGHV036; [Dihydrofolate reductase-thymidylate partial; ORF67, WSSV] | 13.794 | 2 [2] | 19.1 | (BP):dTMP biosynthetic process; pyrimidine nucleobase metabolic process; (MF): thymidylate synthase activity | SP, TM |
| B0YLJ2 | SGHV038 | 136.68 | 32 [32] | 33.9 |  | Coiled coils; SP; RGD motif; α Amylsase catalytic domain (α-β barrel containing active site) |
| B0YLJ3 | SGHV039 | 37.659 | 3 [3] | 8.4 |  | TM; SP; Threonine-rich regions; interrupted by proline/serine residues |
| B0YLJ4 | SGHV040; [ORF AMV130, *Amsacta moorei* EPV] | 104.13 | 3 [3] | 3.7 |  |  |
| B0YLJ5 | SGHV041; [ORF MSV214, *Melanoplus sanguinipes* EPV] | 48.773 | 4 [4] | 9.7 |  | PD-(D/E) XK nuclease fold |
| B0YLJ6 | SGHV042 | 14.248 | 2 [2] | 21.8 |  | Coiled coils; TM helix |
| B0YLJ7 | SGHV043 | 16.899 | 6 [1] | 54.2 |  | SP; Glutamine-rich region |
| B0YLJ8 | SGHV044 | 42.84 | 4 [4] | 13.6 |  | Coiled coils; TM; t-SNAREs; SF3 Helicase; Asparagine-rich region |
| **B0YLJ9** | **SGHV045** | **201.11** | **13 [13]** | **9.1** |  | **PPASE (inorganic pyrophosphatase)** |
| B0YLK0 | SGHV046 | 61.536 | 19 [18] | 43 |  |  |
| B0YLK1 | SGHV047; [Cellular protein (CBG22662)- *Caenorhabditis briggsae*] | 47.187 | 10 [10] | 25.7 |  | NUDIX hydrolase domain-like; Coiled coils; Pre-mRNA Splicing factor 9-like protein |
| B0YLK4 | SGHV050 | 32.741 | 10 [2] | 41.9 |  |  |
| B0YLK7 | SGHV053; [*per-os* infectivity factor 2-like protein; *Gryllus bimaculatus* nudivirus] | 40.239 | 3 [2] | 12.2 |  | SP |
| B0YLL4 | SGHV060 | 24.155 | 2 [2] | 11.1 |  |  |
| B0YLL5 | SGHV061 | 57.433 | 7 [6] | 17.4 |  | TM helix |
| **B0YLL6** | **SGHV062; [ORF147, *Trichoplusia ni ascovirus*-2C[** | **512.12** | **32 [32]** | **9.7** |  | **SP; NLS-BP, Nebulin-repeats; GBD-FH3; Leucine-zipper; EzrA; NUMOD3 motifs; Spectrin repeats; *t*-SNAREs** |
| B0YLL8 | SGHV064; ATP-binding cassette transporter-like protein; [ORF AMV130 *Ambulyx moorei* EPV] | 70.023 | 21 [20] | 37.5 | (MF): ATP binding | Coiled coils; ATP-binding cassette transporter; PUM; Zinc Finger domain |
| B0YLM1 | SGHV067 | 31.019 | 12 [10] | 41.3 |  | TM helix |
| B0YLM2 | SGHV068 | 12.651 | 2 [2] | 11.1 |  | TM helix, SP |
| B0YLM3 | SGHV069 | 30.914 | 9 [9] | 41.1 |  |  |
| B0YLM4 | SGHV070 | 50.921 | 6 [5] | 21.3 |  |  |
| B0YLM6 | SGHV072 | 31.771 | 6 [4] | 21.2 | (MF): Thiol oxidase activity; (BP):oxidation-reduction process | TM; ERV/ALR sulfhydryl oxidase domain (mitochondrial) |
| B0YLN1 | SGHV077; ATP-binding cassette transporter-like protein; [ORF AMV130 *Amsacta moorei* EPV] | 125.83 | 3 [2] | 3.9 | (MF): ATP binding | DNA-directed DNA polymerase, family B, exonuclease domain; Ribonuclease H-like domain |
| B0YLN7 | SGHV083; [ORF AMV214 *Ambulyx moorei* EPV] | 81.59 | 8 [7] | 15.3 |  | NLS-BP |
| B0YLN8 | SGHV084 | 25.768 | 3 [2] | 13.7 |  |  |
| B0YLN9 | SGHV085 | 30.09 | 10 [10] | 38.4 |  | α Helix-β stand-α helix |
| B0YLP0 | SGHV086 | 70.176 | 17 [16] | 30.9 |  | α Helix-β stand-α helix |
| B0YLP2 | SGHV088 | 77.76 | 3 [2] | 4.3 |  | TM helix |
| B0YLP5 | SGHV091 | 31.493 | 2 [2] | 7.5 |  | TM; SP |
| B0YLP7 | SGHV093 | 38.532 | 17 [6] | 50.2 |  | TM |
| B0YLP8 | SGHV094 | 32.7 | 16 [15] | 64.5 |  | TM; α Helix-β stand-α helix |
| B0YLQ0 | SGHV096 | 43.527 | 20 [10] | 59.8 |  | TM helix |
| B0YLQ1 | SGHV097 | 44.403 | 15 [12] | 40.1 |  | TM helix |
| B0YLQ2 | SGHV098 | 13.533 | 6 [6] | 43.5 |  | TM |
| B0YLQ5 | SGHV101 | 12.338 | 4 [4] | 45.3 |  | TM helix |
| B0YLQ6 | SGHV102; [*per-os* infectivity factor-1-like protein; *Neodiprion abietis* NPV] | 76.116 | 6 [5] | 13 |  | TM helix, SP; EGF-like domain; multiple tyrosine kinase phosphorylation sites |
| B0YLQ8 | SGHV104 | 77.885 | 3 [3] | 6.4 |  | TM; Coiled coils |
| B0YLQ9 | SGHV105 | 34.772 | 2 [2] | 7.2 |  | Coiled coils |
| B0YLR0 | SGHV106 | 55.083 | 7 [3] | 25.8 |  | Coiled coils; Serine/Threonine/Glutamine-rich stretches |
| B0YLR1 | SGHV107; AA ATPase central domain protein; Lymphocystis disease virus (China isolate) | 59.564 | 15 [14] | 32.6 | (MF): ATP binding; nucleoside-triphosphatase activity; (BP):metabolic process; cell division | SP; AAA-ATPase Central domain protein; PAN |
| B0YLR2 | SGHV108; cell division protein 48-like protein; Lymphocystis disease virus (China isolate) | 63.944 | 6 [6] | 16 | (MF): ATP binding; nucleoside-triphosphatase activity; (BP):metabolic process; cell division | SP; P-loop /AA-ATPase Central domain protein; PAN |
| B0YLR4 | SGHV110; [Matrix metalloproteinase-28 (mp-nase)-like protein; *Spodoptera litura* GV] | 23.634 | 2 [2] | 11.4 | (CC): extracellular matrix; (MF): metalloendopeptidase activity; zinc ion binding; (BP):proteolysis | TM helix, SP, Zinc-dependent metalloprotease signature peptidase M10; Helix-turn-helix domain |
| B0YLR6 | SGHV112 | 19.057 | 6 [5] | 24 |  | TM helix; SP |
| B0YLR7 | SGHV113; [Protein PY00593, *Plasmodium yoelli yoelli* strain 17XNL] | 33.128 | 8 [8] | 30.6 |  | Cellular protein PY00593, *Plasmodium yoelii yoelii* Str.17XNL |
| **B0YLT3** | **SGHV129** | **9.4087** | **2 [2]** | **43.6** |  | **Coiled coils** |
| B0YLT8 | SGHV134 | 11.781 | 2 [2] | 23.2 |  | Coiled coils |
| B0YLU1 | SGHV137 | 10.618 | 2 [2] | 23.3 |  | Coiled coils |
| B0YLU4 | SGHV140 | 48.44 | 11 [9] | 31.4 |  | Coiled coils |

WSSV, white spot syndrome virus; NPV, nucleopolyhedrosis virus; EPV, entomopoxvirus; GV, granulovirus; BP, Biological process; MF, molecular function; CC, cellular localization; TM, transmembrane domain; SP; signal peptide; RGD, arginyl-glycyl-aspartic acid; EGF, Epidermal growth factor; NLS-BP, bipartite nuclear recognition signal; PPASE-TENSIN, Tensin phosphatase; HDAC, histone deacetylase; PD-(D/E) XK, conserved domain of nuclease superfamily involved in various nucleic acid metabolism; *t*-SNAREs, *t*arget membrane-associated Soluble *N*-ethyl-maleimide-sensitive fusion protein (NSF) Attachment Protein (SNAP) REceptors; SF3-Helicase, superfamily 3 of helicases, assists viruses to initiate their own replication bypassing host's cell-based regulation; NUDIX, a housekeeping enzyme that hydroxylase a NUcleoside DIphosphate linked to another moiety, X; GBD-FH3, GTPase-binding and formin homology 3 domain; EzrA, septation FtsZ-ring formation regulator; NUMOD3, Nuclease-associated modular domain 3; PAN, Proteasome activating nucleotidase.

**Table S 8: Annotation of nine *Wigglesworthia glossinidia* proteins that were detectable in the proteome of GpSGHV-infected *G. m. morsitans***: Four and five *W. glossinidia* proteins were up- and down-regulated, respectively (See also Figure 4).

| **UniProt ID** | **Protein name [Description]** | **Mol. weight [kDa]** | **Peptides** | **Sequence coverage [%]** | **Predicted signature/conserved domains; [amino acid coordinates in the protein sequence]** |
| --- | --- | --- | --- | --- | --- |
| **Up-regulated proteins (*n* = 4)** | | | | | |
| H6Q4L0 | Chaperone protein HtpG | 74.36 | 2 | 2.2 | GHKL domain (Gyrase, Hsp90, Histidine Kinase, MutL) (25-186), Hsp90 (217-635) |
| H6Q4M6 | Phosphoribosylaminoimidazolecarboxamide formyltransferase/IMP cyclohydrolase | 59.563 | 2 | 1.9 | MGS-like domain (20-131) |
| H6Q518 | Chaperone protein DnaK | 70.529 | 2 | 2.7 | HSP70 (4-603) |
| H6Q5R0 | Transcription repair coupling factor (TRCF) protein | 78.971 | 2 | 3.6 | CarD-like/TRCF domain (19-118), DEAD (147-311), Helicase C (375-451), TRCF (550-649) |
| **Down-regulated proteins (n = 5)** | | | | | |
| H6Q489 | Bifunctional n-acetylglucosamine-1-phosphate uridyl-transferase glucosamine-1-phosphate acetyltransferase (GlmU) | 50.888 | 2 | 3.1 | MobA-like NTP transferase domain (10-152), Hexapep (272-435) |
| H6Q4P5 | Potassium transporter (TrK) protein | 51.392 | 2 | 7.4 | TrkA-N domain (3-125; 236-351), TrkA-C domain (160-227) |
| H6Q549 | Exonuclease V (RecBCD complex) β-subunit | 137.82 | 2 | 2 | UvrD/REP helicase N-terminal domain (3-427), UvrD C (430-807) |
| H6Q560 | DNA polymerase I (pol A) | 31.649 | 2 | 10.3 | 5'-3' exonuclease, N-terminal resolvase-like domain (6-168), 5'-3' exonuclease, C-terminal SAM fold (170-272) |
| H6Q5Y8 | Protein TolA | 29.185 | 2 | 8.4 | TolA (155-247) |

TrK, NAD-binding component of potassium transporter; GlmU, Bi-functional protein GlmU; RecBCD, Exonuclease V β-subunit; TolA, TolA family protein; polA, DNA polymerase I; HtpG, Chaperone protein HtpG; purH, Phosphoribosyl-aminoimidazole-carboxamide formyl transferase/IMP cyclohydrolase; TRCP, transcription repair coupling factor; DnaK, Chaperone protein DnaK.
